# 4-1BB stimulation with concomitant inactivation of adenosine A2B receptors enhances CD8^+^ T cell antitumor response

**DOI:** 10.1172/JCI190841

**Published:** 2025-04-03

**Authors:** Jihae Ahn, Ping Xie, Siqi Chen, Guilan Shi, Jie Fan, Minghui Zhang, Hui Tang, Amanda R. Zuckerman, Deyu Fang, Yong Wan, Timothy M. Kuzel, Yi Zhang, Bin Zhang

**Affiliations:** 1 Department of Medicine, Hematology/Oncology Division, Robert H. Lurie Comprehensive Cancer Center, and; 2 Department of Pathology, Northwestern University Feinberg School of Medicine, Chicago, Illinois, USA.; 3 Department of Pharmacology and Chemical Biology, Emory University School of Medicine, Atlanta, Georgia, USA.; 4 Biotherapy Center, The First Affiliated Hospital of Zhengzhou University, Zhengzhou, Henan, China.

**Keywords:** Immunology, Oncology, Adaptive immunity, Immunotherapy, T cells

## Abstract

Activating the immune costimulatory receptor 4-1BB (CD137) with agonist antibody binding and crosslinking-inducing agents that elicit 4-1BB intracellular signaling potentiates the antitumor responses of CD8^+^ T cells. However, the underlying in-depth mechanisms remain to be defined. Here, we show that agonistic 4-1BB treatment of activated CD8^+^ T cells under continuous antigenic stimulation makes them more metabolically vulnerable to redox perturbation by ablation of intracellular glutathione (GSH) and glutathione peroxidase 4 (GPX4) inhibition. Further, genetic deletion of adenosine A2B receptor (A2BR) induces superior survival and expansion advantage of competent CD8^+^ T cells with agonistic 4-1BB costimulation, leading to more effective antitumor efficacy of adoptive cell therapy (ACT). Mechanistically, A2BR deletion helps sustain the increased energy and biosynthetic requirements through the GSH/GPX4 axis upon 4-1BB costimulation. A2BR deletion in combination with agonistic 4-1BB costimulation displays a greater ability to promote antitumor CD8^+^ effector T cell survival and expansion while mitigating T cell exhaustion. Thus, the A2BR pathway plays an important role in metabolic reprogramming with potentiation of the GSH/GPX4 cascade upon agonistic 4-1BB costimulation that allows the fine-tuning of the antitumor responses of CD8^+^ T cells.

## Abstract 2

<span>Targeting A2BR potentiates </span><span>4-1BB costimulation-mediated CD8^+^ T cell antitumor responses, which relies on sustaining the GSH-GPX4 cascade.</span>

## Graphical abstract

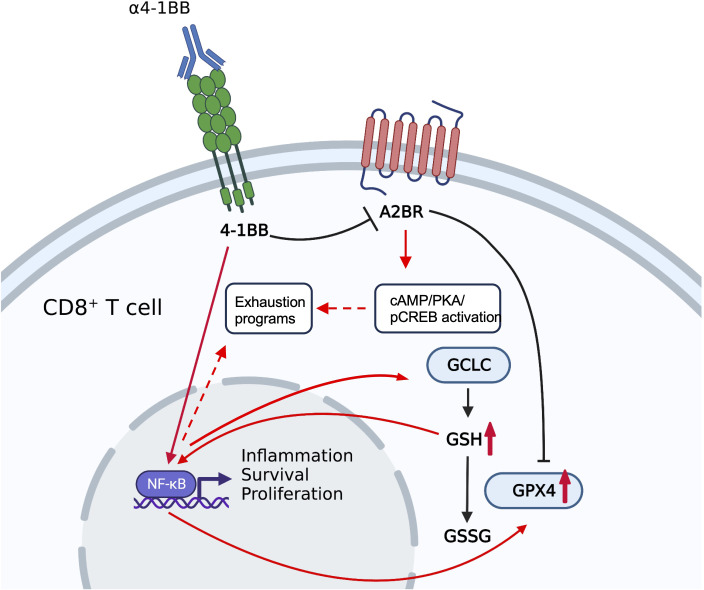

## Introduction

Although checkpoint inhibitors targeting PD-1/PD-L1 and/or CTLA-4 T cell coinhibitory receptors have revolutionized cancer treatment and rejuvenated the field of immuno-oncology, immunostimulatory agonist mAbs targeting costimulatory receptors, including members of the TNF receptor superfamily (TNFRSF) such as 4-1BB (CD137), have gained great momentum in clinical development for cancer immunotherapy beyond their successful application in chimeric antigen receptor (CAR) T cell therapy (1–3). 4-1BB is highly expressed on activated CD4^+^ and CD8^+^ T cells (4) and recruits the TRAF-1/2 signaling adaptors (5, 6) to stimulate downstream activation of the NF-κB transcriptional pathway upon engagement with its cognate ligand (7). Activation of CD137 induces potent costimulatory signals to CD8^+^ cytotoxic T lymphocytes (CTLs), promoting cell proliferation and survival, as well as differentiation into memory cells (8). Consistent with its costimulatory activity, agonistic mAbs against CD137 have mounted effective antitumor effects in multiple preclinical models (9–11) that have been mainly attributed to the reinvigoration of dysfunctional CD8^+^ tumor-infiltrating lymphocytes (TILs) (12, 13). Earlier studies have shown that agonistic anti-4-1BB treatment activates glucose and fatty acid metabolism to support CD8^+^ T cell proliferation (14). Recent studies further suggest that 4-1BB costimulation induces T cell mitochondrial biogenesis and improves metabolism to elicit cancer immunotherapeutic responses (15), in line with the roles of the 4-1BB–signaling domain to sustain the longevity and memory capacity of CAR T cells, resulting in enhanced antitumor potential (16, 17). However, the main metabolic axis that 4-1BB signaling utilizes to promote CTLs and the potential consequences for adoptive cell therapy (ACT) remain poorly defined.

We and others have previously demonstrated a critical role of the adenosine-producing ectoenzymes ecto-nucleoside triphosphate diphosphohydrolase-1 (NTPDase1/CD39) and ecto-5′-nucleotidase/CD73 (NT5E) in tumor immune escape (18–21). Mechanistically, adenosine generated from the CD73/CD39 axis mitigates antitumor CTL responses mainly by binding the G-protein–coupled type 2 adenosine receptors A2A/A2B (A2AR/A2BR). Interestingly, we have shown that agonistic anti–4-1BB treatment preferentially promotes CD73^–^ effector CD8^+^ T cell response to inhibit tumor growth and progression. Anti-CD73 neutralizing mAb further improves ant–4-1BB therapy associated with enhanced antitumor T cell immunity (22), indicating the involvement of CD73-mediated adenosinergic action in the control of targeting of 4-1BB. On the other hand, studies have revealed that A2AR activation impairs metabolic activity of both oxidative phosphorylation (OXPHOS) and glycolysis in activated CD8^+^ T cells (23). We have recently demonstrated a key role of glutathione (GSH)/glutathione peroxidase 4 (GPX4) and adenosinergic pathways in fine-tuning the metabolic fitness of antitumor CD8^+^ T cells (24). It remains unclear, however, whether and how the relevant type 2 adenosine receptor activation contributes to metabolic fitness and functional competence of CD8^+^ T cells upon 4-1BB costimulation.

Here, we show that activated CD8^+^ T cells with 4-1BB costimulation display a distinct phenotypic, transcriptomic, and metabolic profile toward GSH biosynthesis and utilization by GPX4 under continuous antigenic stimulation. Agonistic anti–4-1BB treatment elicits its immunostimulatory function on CD8^+^ T cells through engagement of NF-κB–dependent transcriptional modulation of GPX4. Further, 4-1BB costimulation downregulates expression of A2BR but not A2AR, and A2BR deletion potentiates the GSH/GPX4 metabolic cascade, leading to improved CD8^+^ T cell mitochondrial fitness and functional competence upon treatment with agonistic antibodies against 4-1BB (α4-1BB). Consequently, A2BR deletion enhances the expansion of tumor-specific CD8^+^ T cells and protects them from an exhausted-like phenotype, leading to superior antitumor effects of ACT in combination with agonistic α4-1BB treatment. Together, these data reveal that 4-1BB costimulation relies on the GSH/GPX4 metabolic axis involving A2BR regulation in concordance with its well-established functions in survival, expansion, and differentiation, thereby increasing competent CD8^+^ T cell persistence by A2BR deletion.

## Results

### Increased intracellular levels of GSH and GPX4 in 4-1BB costimulation of activated CD8^+^ T cells.

**Figure 1:**
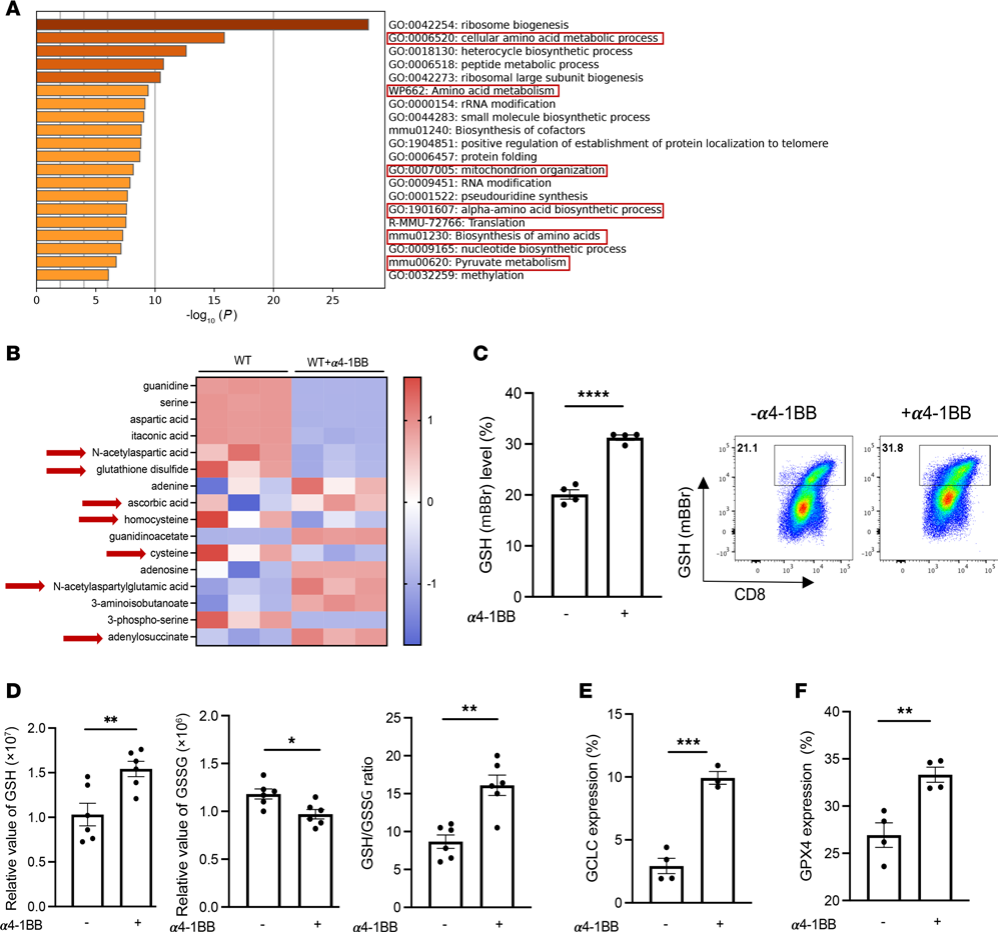
4-1BB costimulation promotes intracellular accumulation of GSH and GPX4 in activated CD8^+^ T cells. (**A**) Bulk RNA-Seq was performed in activated CD8^+^ T cells stimulated by anti-CD3 and anti-CD28 with or without α4-1BB (clone 3H3, 100 ng/ml) for 4 days. The enriched pathways are shown. (**B**) Metabolic profiling was performed by LC-MS in activated CD8^+^ T cells treated as above with or without α4-1BB. Among the top changes in metabolite composition, 7 metabolites involved in the GSH-related pathway in α4-1BB–treated cells are highlighted with red arrows. (**C**) Intracellular GSH content of activated CD8^+^ T cells was measured by mBBr staining using flow cytometry. (**D**) Relative levels of GSH and GSSG were determined by LC-MS, and the GSH/GSSG ratio was calculated. Expression levels of GCLC (**E**) and GPX4 (**F**) were measured by flow cytometry. Data (means ± SEM) are representative of 3 (**C**), 2 (**D**) or 5 (**E** and **F**) independent experiments. (**C**–**F**) Unpaired Student’s *t* test. *P < 0.05; **P < 0.01; ***P < 0.001; ****P < 0.0001.

To search for the potential mechanism by which 4-1BB costimulation promotes CD8^+^ T cell responses, we performed a global transcriptome analysis by RNA-Seq. The pathway enrichment analysis revealed that pathways related to cellular metabolism, such as amino acid biogenesis and metabolism, including mitochondria organization for metabolic processes oxidative stress and redox, were among the top enrichment in response to α4-1BB in murine-activated CD8^+^ T cells in vitro (Figure 1A). Given the emerging role of T cell metabolism in immunotherapy (25), particularly in the context of 4-1BB costimulation (14, 15), we explored subsequently how 4-1BB stimulation is primarily linked to T cell metabolism through metabolic profiling via liquid chromatography–mass spectrometry (LC-MS) on murine CD8^+^ T cells with and without α4-1BB treatment. Among the top changes in metabolite composition, 7 metabolites (26–32) were involved in the GSH-related redox pathway in α4-1BB-treated cells (Figure 1B). Notably, α4-1BB treatment resulted in an increase in GSH or glutamate that is used to synthesize GSH, but a decrease in glutathione disulfide (GSSG), an oxidized form of GSH (Figure 1B). In line with these results, increased levels of intracellular GSH were confirmed by flow cytometry using monobromobimane (mBBr) dye (Figure 1C). Likewise, α4-1BB treatment augmented the intracellular GSH content, leading to an increased ratio of GSH/GSSG (Figure 1D). The α4-1BB–mediated GSH increase was associated with an elevated level of glutamate-cysteine ligase catalytic subunit (GCLC) (Figure 1E), the first rate-limiting enzyme of GSH de novo synthesis (33, 34). We also found that 4-1BB agonism promoted an increase in GPX4 (Figure 1F) that is capable of utilizing GSH to detoxify lipid peroxidation (35, 36). In addition, we confirmed the ability of 4-1BB agonism to increase GSH (Supplemental Figure 1A; supplemental material available online with this article; https://doi.org/10.1172/JCI190841DS1) and GPX4 (Supplemental Figure 1B) in a dose-dependent manner, which was consistent with the ability of 4-1BB agonism to promote T cell proliferation determined by Ki-67 expression (Supplemental Figure 1C).

### Enhancement of CD8^+^ T cell function by α4-1BB treatment requires intracellular GSH.

**Figure 2:**
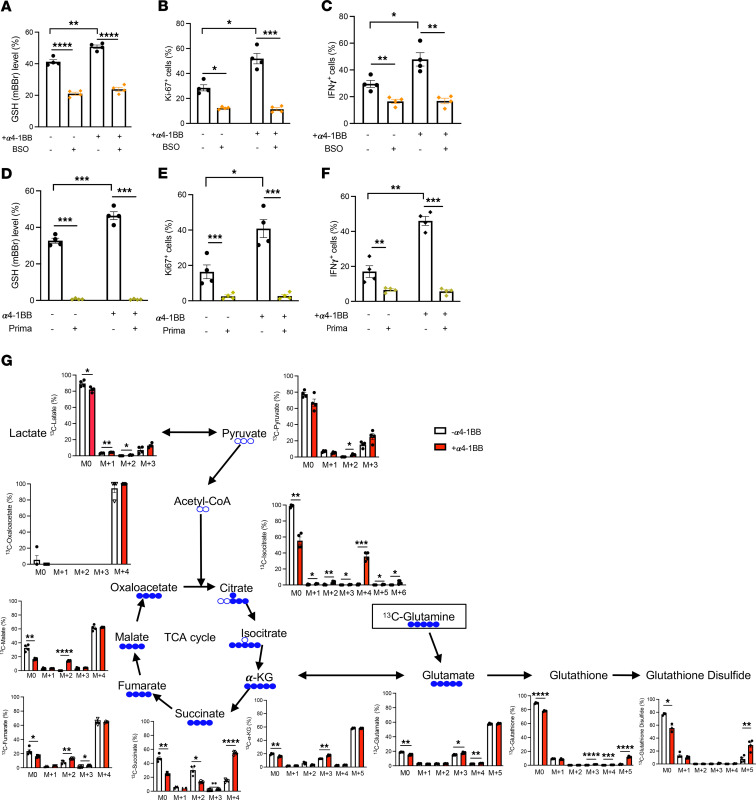
Intracellular GSH content is required for α4-1BB–mediated enhancement in CD8^+^ T cell function. Pmel CD8^+^ T cells were stimulated by anti-CD3 and anti-CD28 in the presence or absence of α4-1BB (clone 3H3, 100 ng/ml) with or without ablation of intracellular GSH by either BSO or Prima-1^met^ for 4 days. Intracellular GSH content (**A** and **D**), expression of Ki-67 (**B** and **E**), and IFN-γ^+^ cells (**C** and **F**) were measured by flow cytometry. (**G**) The LC-MS analysis of incorporation of [U-^13^C] glutamine-derived carbons into individual metabolites involving TCA cycle and GSH synthesis after stable isotope labeling with [U-^13^C] glutamine. M0, unlabeled mass of isotope; M+n, native metabolite mass (M) plus number of isotopically labeled carbons (*n*). The solid blue balls represent ^13^C carbons, and the empty balls represent ^12^C carbons. Data (means ± SEM) are representative of 2 (**A**–**F**) independent experiments. Two-way ANOVA with Bonferroni’s post test correction was used. *P < 0.05; **P < 0.01; ***P < 0.001; ****P < 0.0001.

To determine the importance of GSH for CD8^+^ T cell activities stimulated with anti-CD3/CD28 in the presence or absence of 4-1BB agonism, we added buthionine sulfoximine (BSO) to block de novo GSH synthesis or Prima-1^met^ to deplete intracellular GSH. Consistent with previous studies (37, 38), ablation of intracellular GSH by either BSO or Prima-1^met^ (Figure 2, A and D) diminished Ki-67 expression (Figure 2, B and E) and IFN-γ production (Figure 2, C and F) in activated CD8^+^ T cells stimulated with anti-CD3/CD28. Likewise, addition of 4-1BB costimulation failed to promote CD8^+^ T cell responses upon ablation of intracellular GSH by either BSO or Prima-1^met^ (Figure 2, A–F), suggesting a crucial role of intracellular GSH content for α4-1BB–mediated enhancement in CD8^+^ T cell function. We next sought to determine the metabolic consequence(s) of α4-1BB treatment in GSH biosynthesis in activated CD8^+^ T cells using [U-^13^C] glutamine as an isotope tracer. We measured the contributions of labeled glutamine to total de novo GSH synthesis by quantifying ^13^C-labeled reduced (GSH) plus oxidized GSH (GSSG) levels derived from glutamine tracers (Figure 2G). Glutamine carbons were more readily incorporated into the glutamate moiety of the GSH upon α4-1BB treatment, confirming that glutamine is one of the main precursors for GSH synthesis. Furthermore, the ^13^C fractional distribution in GSSG isotopologues (m+5) was higher, which could reflect a concomitant oxidation of de novo–synthesized GSH in response to 4-1BB costimulation. It is also likely that the increased ratio of GSH/GSSG upon 4-1BB agonism contributes to reduced oxidative stress and improved metabolic fitness in activated CD8^+^ T cells. In addition, the m+3 α-ketoglutarate (α-KG) from labeled glutamine was elevated upon α4-1BB treatment. The increased synthesis of m+4 succinate, m+2 fumarate, and m+2 malate is consistent with the oxidation of labeled α-KG via the forward reactions of the tricarboxylic acid (TCA) cycle. The pattern of glutamine carbons entering the TCA cycle in α4-1BB–treated T cells — including increased labeled TCA intermediates (Figure 2G) — is indicative of enhanced TCA cycle activity. There was also a marked level of unlabeled and ^13^C4-isocitrate (m+4) upon 4-1BB costimulation, suggesting a source or sources of unlabeled acetyl-CoA that contribute to the continued operation of the TCA cycle. Taken together, these data show that α4-1BB treatment caused a substantial increase in [^13^C] glutamine-derived carbon incorporation into GSH synthesis and utilization while more effectively fueling each step in the TCA cycle, which supports cellular bioenergetics for cell survival and proliferation upon 4-1BB costimulation.

### Enhanced reliance on GPX4 is essential to improve activated CD8^+^ T cell functions by 4-1BB agonism.

**Figure 3:**
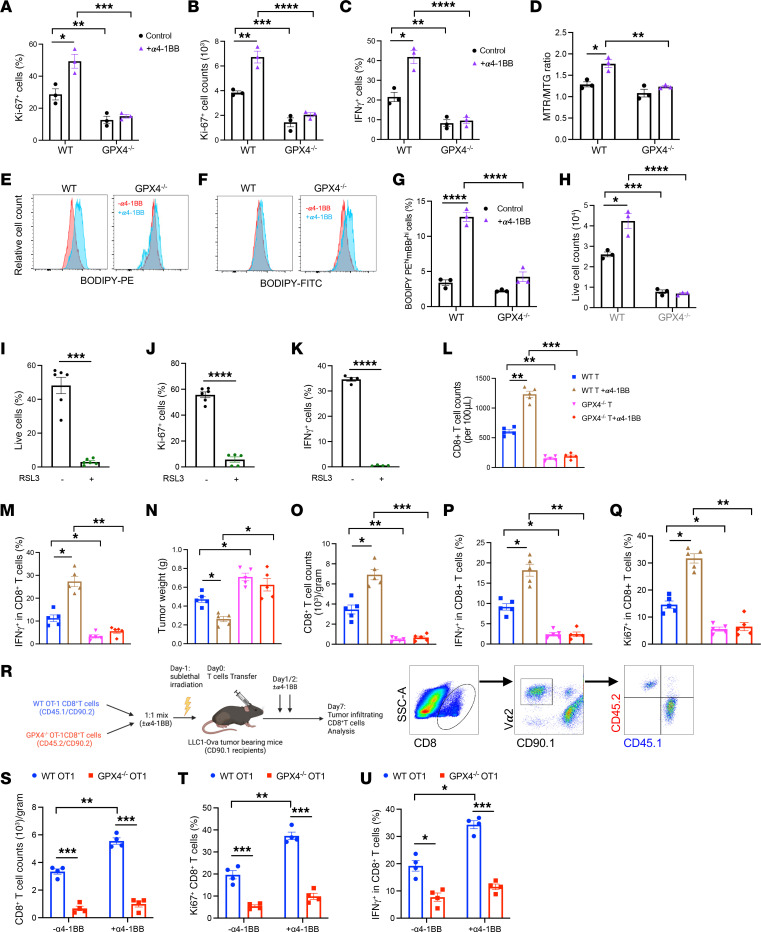
GPX4 is essential for α4-1BB–mediated enhancement in CD8^+^ T cell function. Ki-67 expression (**A**), Ki-67^+^ cells (**B**), IFN-γ^+^ cells (**C**) the ratio of MTR/MTG (**D**), expression levels of BODIPY-PE (**E**), BODIPY-FITC (**F**), BODIPY-PE^hi^mBBr^hi^ cells (**G**), and viable cell counts (**H**) were determined in treated CD8^+^ T cells by flow cytometry. Frequency of viable cells (annexin V^–^/7-AAD^–^) (**I**), Ki-67^+^ cells (**J**), and IFN-γ^+^ cells (**K**) were determined in treated CD8^+^ T cells in the absence or presence of the GPX4 inhibitor RSL3. Following conditioning with a sublethal irradiation dose (600 cGy), MC38 tumor–bearing CD90.1^+^ mice (*n* = 4–5) received polyclonal tumor-reactive CD90.2^+^ WT or GPX4^–/–^ CD8^+^ T cells and were treated further with or without α4-1BB (100 μg/mouse) by i.p. daily once for 2 days. Seven days after T cell transfer, viable cell counts (**L**), IFN-γ^+^ cell counts (**M**) in peripheral blood by flow cytometry, tumor weight (**N**), transferred CD8^+^ T cell counts (**O**), frequency of IFN-γ^+^ cells (**P**) and Ki67^+^ cells (**Q**) among transferred CD8^+^ T cells were determined. For the T cell adoptive transfer in a LLC1-Ova tumor model (**R**), seven days after OT-I T cell transfer, relative counts of total transferred CD8^+^ T cells (**S**) and frequency of Ki-67^+^CD8^+^ cells (**T**), and IFN-γ^+^CD8^+^ cells (**U**) among transferred tumor-infiltrating T cells were determined by flow cytometry. Data (means ± SEM) are representative of 2–3 (**A**–**H**) or 2 (**I**–**K** and **R**–**U**) independent experiments. (**I**–**K**) Unpaired Student’s *t* test. (**A**–**H** and **L**–**U**) Two-way ANOVA with Bonferroni’s post test correction was used. *P < 0.05; **P < 0.01; ***P < 0.001; ****P < 0.0001.

It has been recently reported that optimal CD8^+^ T cell function requires GPX4 (39). As α4-1BB increased simultaneous expression levels of GSH and GPX4, we wanted to examine how GPX4 ablation affected CD8^+^ T cell function with or without 4-1BB agonism. While α4-1BB increased Ki-67 expression (Figure 3A) and induced more proliferating Ki-67^+^ WT T cells (Figure 3B), this α4-1BB–mediated activity was completely abolished in GPX4^–/–^ CD8^+^ T cells (Figure 3, A and B). Furthermore, the frequency of IFN-γ^+^ cells was significantly elevated in WT but not GPX4^–/–^ CD8^+^ T cells in response to α4-1BB treatment (Figure 3C). Interestingly, 4-1BB costimulation of WT T cells resulted in an increased ratio of mitochondria membrane potential (MitoTracker red [MTR]) over mitochondria mass (MitoTracker green [MTG]) (Figure 3D) suggestive of improved mitochondrial function, in line with previous studies (40). By contrast, GPX4^–/–^ CD8^+^ T cells with 4-1BB agonism presented a significant decrease in the ratio of MTR/MTG consistent with mitochondrial loss of function compared with WT counterparts (Figure 3D). Among the family members of GPX that catalyze the reduction of hydrogen and lipid peroxides, only GPX4 directly detoxifies lipid hydroperoxides within biological membranes (41). GPX4 is thus an essential regulator of ferroptosis, which is a nonapoptotic form of cell death by accumulation of ROS (35, 36). We next examined the effect of 4-1BB agonism on lipid peroxidization between WT and GPX4^–/–^ CD8^+^ T cells. We utilized the BODIPY 581/591 dye to determine cellular lipid oxidation based on a shift of the fluorescence emission peak from –590 nm (red, phycoerythrin [PE] channel) to –510 nm (green, FITC channel). 4-1BB costimulation resulted in an increase of BODIPY-PE in WT but not GPX4^–/–^ CD8^+^ T cells (Figure 3E). By contrast, 4-1BB costimulation resulted in an increase of BODIPY-FITC in GPX4^–/–^ but not WT CD8^+^ T cells (Figure 3F). These results suggest that the presence of GPX4 is likely critical in rendering activated CD8^+^ T cells resistant to lipid oxidation upon 4-1BB agonism. Moreover, combining with the mBBr staining that measures intracellular GSH content, the portion of BODIPY-PE^hi^mBBr^hi^ cells could be thus indicative potentially for activated cells resistant to ferroptosis. Indeed, the frequency of BODIPY-PE^hi^mBBr^hi^ was significantly increased in the activated WT (Supplemental Figure 1D), but not GPX4^–/–^ CD8^+^ T cells with α4-1BB treatment (Figure 3G). As a result, the viable counts were reduced greatly in GPX4^–/–^ CD8^+^ T cells compared with WT T cells, especially with 4-1BB agonism (Figure 3H). To verify the essence of GPX4 for α4-1BB–induced T cell stimulation, activated T cells were treated with or without RAS-selective lethal 3 (RSL3), a well-known inhibitor of GPX4, which effectively induces oxidative cell death through ferroptosis. Similar to the results obtained from GPX4^–/–^ CD8^+^ T cells, RSL3 treatment resulted in a loss of viability (Figure 3I), proliferation (Figure 3J), and IFN-γ production (Figure 3K) for activated CD8^+^ T cells even with 4-1BB agonism.

We next examined whether GPX4^–/–^ CD8^+^ T cells were responsive to α4-1BB treatment in tumor-bearing mice in an adoptive transfer system. Following conditioning with a sublethal irradiation dose, MC38 tumor-bearing CD90.1^+^ mice received tumor-reactive polyclonal CD90.2^+^ WT or GPX4^–/–^ CD8^+^ T cells followed by treatment further with or without α4-1BB. Consistent with what we observed in vitro, α4-1BB treatment failed to induce accumulation (Figure 3L) and IFN-γ production (Figure 3M) of transferred circulating GPX4^–/–^ CD8^+^ T cells rather than circulating WT T cells. In line with the results we observed previously (24), tumor-reactive GPX4^–/–^ CD8^+^ T cells lost their antitumor activity (Figure 3N) with fewer tumor-infiltrating transferred CD8^+^ T cells (Figure 3O) and decreased IFN-γ production (Figure 3P) and Ki67 expression (Figure 3Q) compared with control CD8^+^ T cells. Notably, α4-1BB treatment facilitated antitumor effects of tumor-reactive WT CD8^+^ T cells, but not those of GPX4^–/–^ CD8^+^ T cells (Figure 3, N–Q). To assess further how GPX4 ablation affected α4-1BB–mediated tumor antigen–specific CD8^+^ T cell responses in vivo, preactivated OT-1 WT (CD45.1^+^) and GPX4^–/–^ (CD45.2^+^) CD8^+^ T cells congenic for CD90.2 were mixed at a 1:1 ratio and cotransferred into LLC1-Ova tumor–bearing recipients (CD90.1^+^) followed with or without α4-1BB treatment. Donor-derived specific CD8^+^ TILs were analyzed 7 days after transfer (Figure 3R). The transferred tumor-infiltrating OT-I GPX4^–/–^ CD8^+^ T cell population was significantly reduced compared with WT counterpart in tumor accumulation (Figure 3S) and lost their ability to proliferate (Figure 3T) and produce IFN-γ (Figure 3U), especially with α4-1BB treatment. In addition, α4-1BB treatment resulted in a significantly increased frequency of exhausted-like PD-1^+^CD39^+^ cells in infiltrating OT-I WT CD8^+^ T cells but not in the case of OT-I GPX4^–/–^ CD8^+^ T cells (Supplemental Figure 2). Collectively these data confirm that GPX4 is crucial to preserving survival, proliferation, and effector functions of activated CD8^+^ T cells potentiated by 4-1BB agonism.

### Crosstalk between the GSH-GPX4 axis and the NF-κB pathway in α4-1BB–mediated CD8^+^ T cell responses.

**Figure 4:**
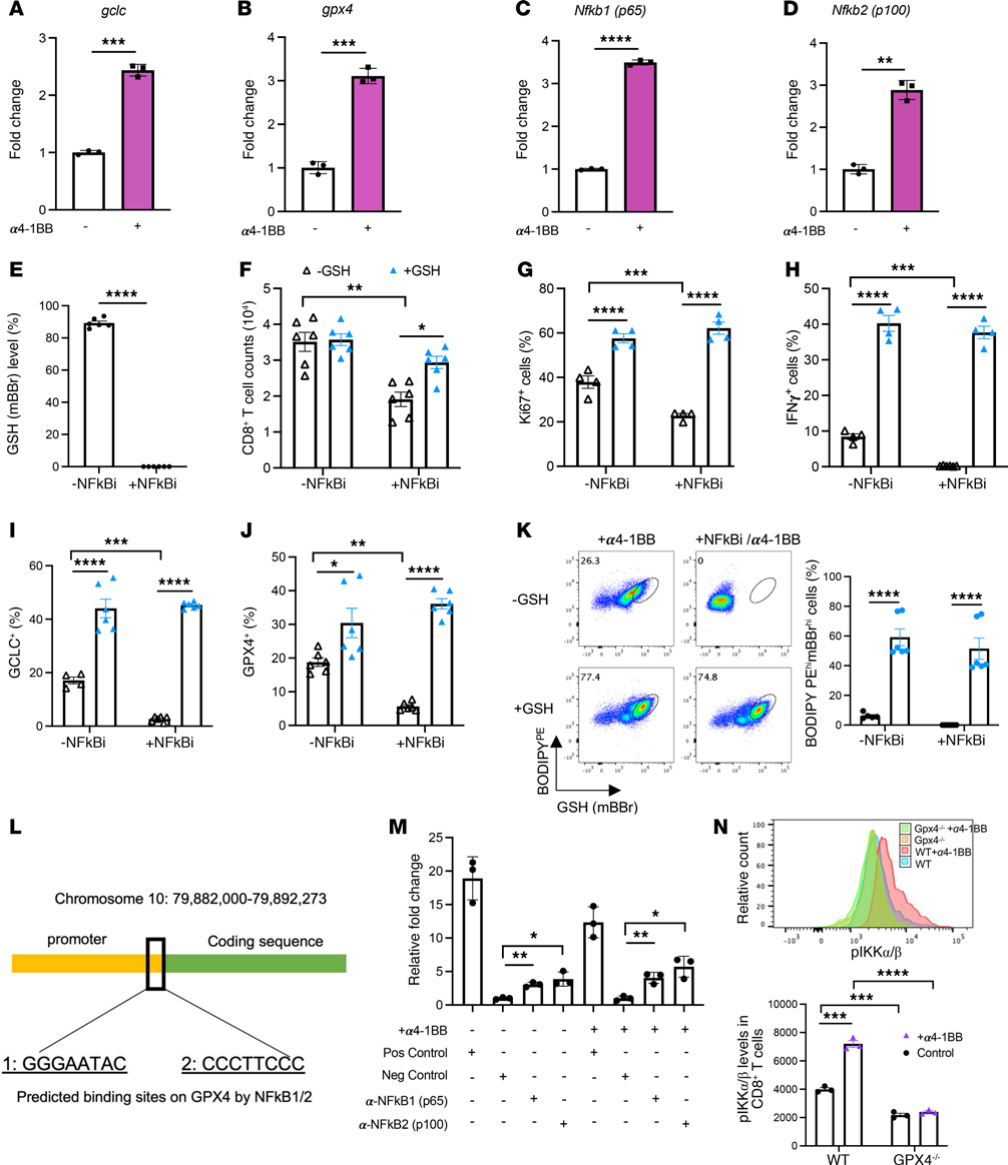
NF-κB–dependent transcriptional modulation of gpx4 expression involving GSH biosynthesis and utilization in potentiating CD8^+^ T cell responses by 4-1BB agonism. CD8^+^ T cells were stimulated by anti-CD3 and anti-CD28 with or without α4-1BB (clone 3H3, 100 ng/ml). Expression levels of *gclc* (**A**), *gpx4* (**B**), *NFkb1* (**C**), and *Nfkb2* (**D**) transcripts were determined by quantitative reverse-transcription PCR (qRT-PCR). The NF-κB inhibitors (Bay 11-7082) and/or cell-permeable GSH was added to α4-1BB–treated CD8^+^ T cell cultures. Intracellular GSH levels (**E**), viable cell counts (**F**), Ki-67^+^ cells (**G**), IFN-γ^+^ cells (**H**), GCLC^+^ cells (**I**), GPX4^+^ cells (**J**), and BODIPY-PE^hi^mBBr^hi^ cells (**K**) were analyzed by flow cytometry. NF-κB–binding sites on the GPX4 promoter were predicted by PROMO-ALGGEN (**L**) and Chip-qPCR analysis confirming the enhanced binding capacity of NF-κb1 and NF-κb2 on *gpx4* in α4-1BB–treated CD8^+^ T cells compared with control cells (**M**). (**N**) Flow cytometric analysis of p-IKKα/β (Ser176/180) levels in WT and GPX4^–/–^ CD8^+^ T cells treated with anti-CD3 and anti-CD28 with or without α4-1BB (clone 3H3, 100 ng/ml) for 24 hours. Data (means ± SEM) are representative of 2 (**A**–**K** and **N**) independent experiments. (**A**–**E**) Unpaired Student’s *t* test. (**F**–**K**, **M**, and **N**) Two-way ANOVA with Bonferroni’s post test correction was used. *P < 0.05; **P < 0.01; ***P < 0.001; ****P < 0.0001.

The NF-κB pathway is a key cell signaling pathway that is rapidly engaged following TCR/CD3, CD28, and TNFR superfamily activation, such as 4-1BB costimulation, that dictates CD8^+^ T cell persistence and effector function (2). We thus wanted to study the connection between the GSH/GPX4 metabolic axis and the NF-κB pathway in activated CD8^+^ T cells with or without 4-1BB costimulation. α4-1BB–treated CD8^+^ T cells showed increased transcripts of *glcl* (Figure 4A) and *gpx4* (Figure 4B) together with *nfkb1* (Figure 4C) and *nfkb2* (Figure 4D). Strikingly, addition of a specific NF-κB inhibitor completely depleted intracellular GSH (Figure 4E), which was associated with significant decreases in viable cellular counts (Figure 4F), proliferation (Figure 4G), and IFN-γ production (Figure 4H) in activated CD8^+^ T cells upon 4-1BB costimulation. Interestingly, addition of cell-permeable GSH promoted further the stimulatory effect of 4-1BB agonism on CD8^+^ T cells in the absence of NF-κB inhibitors (Figure 4, F–H). Furthermore, the impaired cellular functions by NF-κB inhibition were fully restored after addition of cell-permeable GSH (Figure 4, F–H), suggesting the importance of GSH for NF-κB downstream signaling upon 4-1BB costimulation. These CD8^+^ T cells with the addition of cell-permeable GSH also displayed elevated levels of GCLC (Figure 4I) and GPX4 (Figure 4J) accompanying an increase in the frequency of BODIPY-PE^hi^mBBr^hi^ cells (Figure 4K). Similar results were also observed in activated CD8^+^ T cells without 4-1BB costimulation (Supplemental Figure 3, A–G), confirming the fundamental role of the GSH/GPX4 axis in regulating effector T cell accumulation and function through NF-κB signaling. In addition, we examined the direct interaction between NF-κB and *gpx4* in activated CD8^+^ T cells by ChIP-qPCR (ChIP–quantitative PCR) analysis. The NF-κB–binding sites were predicted by PROMO-ALGGEN on the GPX4 promoter (Figure 4L). We validated the binding capacity of both subunits of nuclear factor κ B subunit 1 (NF-κB1) and NF-κB2 on *gpx4* promoter sites upon 4-1BB costimulation (Figure 4M and Supplemental Figure 3H) using 2 specific primers. As the IKK kinase complex is the core regulator of the NF-κB cascade (42), we subsequently measured the phospho-IKKα/β (p-IKKα/β) (Ser176/180) levels in activated WT and GPX4^–/–^ CD8^+^ T cells with or without α4-1BB treatment (Figure 4N). Interestingly, GPX4^–/–^ CD8^+^ T cells exhibited lower levels of p-IKKα/β compared with WT counterparts. Further, 4-1BB–mediated upregulation of p-IKKα/β expression was diminished completely in GPX4^–/–^ CD8^+^ T cells, indicating a GPX4-dependent involvement of activation of the NF-κB signaling pathway upon 4-1BB costimulation. These data together suggest a pivotal role of NF-κB–dependent transcriptional modulation of *gpx4* expression involving GSH biosynthesis and utilization in potentiating CD8^+^ T cell responses by 4-1BB agonism.

### A2BR deletion sustained the GSH/GPX4 axis and promoted CD8^+^ T cell responses upon α4-1BB treatment.

**Figure 5:**
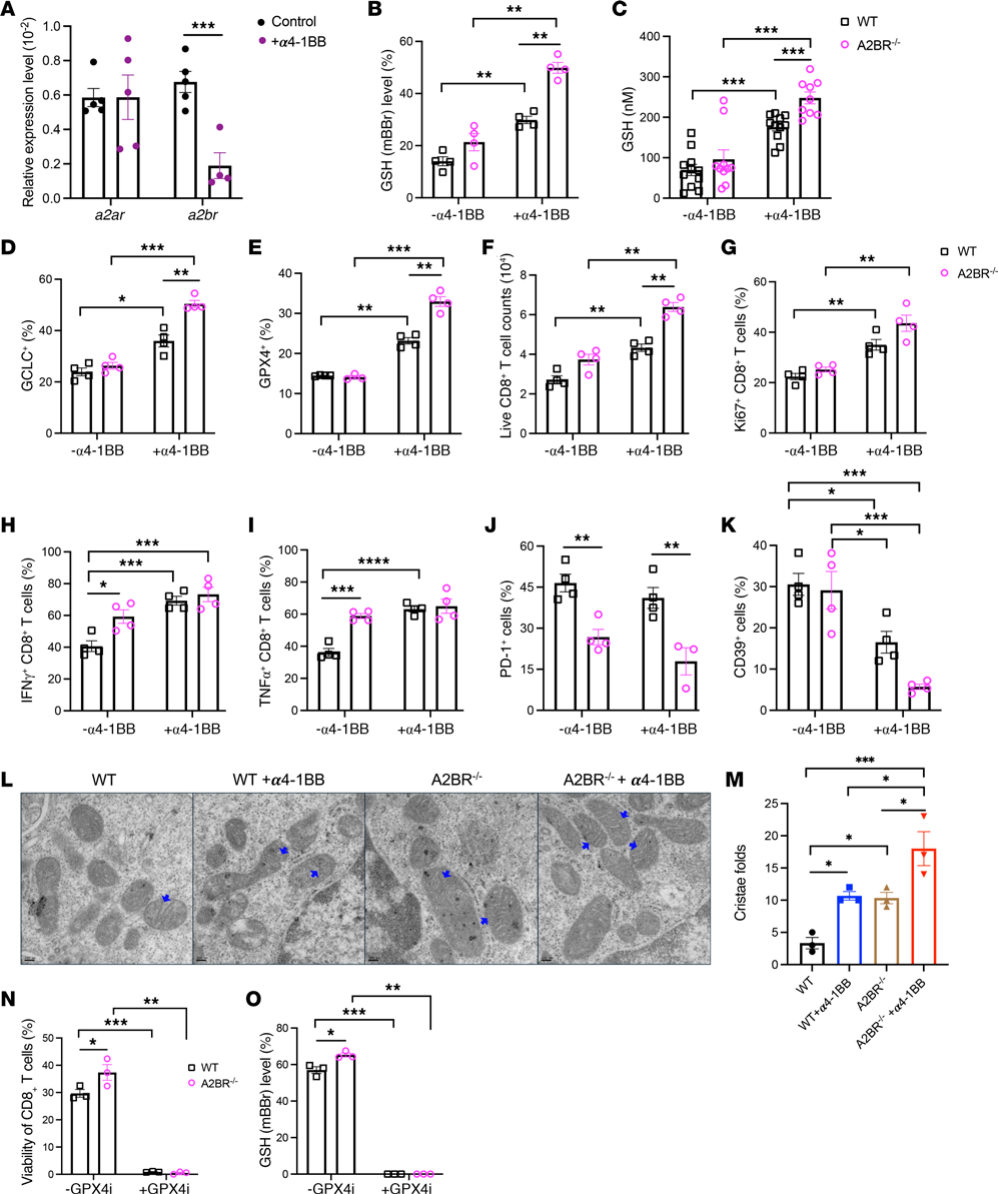
A2BR deletion sustained the GSH/GPX4 axis and promoted CD8^+^ T cell responses upon α4-1BB treatment. CD8^+^ T cells were stimulated by anti-CD3 and anti-CD28 with or without α4-1BB (clone 3H3, 100 ng/ml) for 24 hours. (**A**) Expression levels of *a2ar*, and *a2br* transcripts were determined by qRT-PCR. WT or A2BR^–/–^ CD8^+^ T cells were stimulated by anti-CD3 and anti-CD28 with or without α4-1BB (clone 3H3, 100 ng/ml) for 4 days. Intracellular GSH content was measured by mBBr staining (**B**) and a GSH/GSSG-Glo Assay Kit (**C**). GCLC^+^ cells (**D**), GPX4^+^ cells (**E**), viability (**F**), Ki-67^+^ cells (**G**), IFN-γ^+^ cells (**H**), TNF-α^+^ cells (**I**), PD-1^+^ cells (**J**), and CD39^+^ cells (**K**) were determined by flow cytometry. Transmission electron microscopy analysis on mitochondria (**L**) and cristae folds counts (**M**) in treated CD8^+^ T cells (*n* = 3); original magnification ×10,000. Blue arrows indicate mitochondrial cristae folds. (**N** and **O**) WT or A2BR^–/–^ CD8^+^ T cells were stimulated by anti-CD3 and anti-CD28 with α4-1BB (clone 3H3, 100 ng/ml) in the presence or absence of the GPX4 inhibitor RSL3 for 4 days. Viability (**N**) and GSH levels (**O**) were determined by flow cytometry. Data (means ± SEM) are representative of 2–3 (**B**–**M**) independent experiments. (**A**–**K** and **M**–**P**) Two-way ANOVA with Bonferroni’s post test correction was used. *P < 0.05; **P < 0.01; ***P < 0.001; ****P < 0.0001.

We have previously reported an inhibitory role of the ecto-enzyme CD73–mediated adenosinergic cascade for agonistic anti-4-1BB therapy. To explore further the underlying mechanism, we measured the effects of genetic deletion of the type 2 adenosine receptors including A2AR and A2BR that are the predominant subtypes found on T cells. We utilized an in vitro system (24) whereby transgenic or polyclonal CD8^+^ T cells were expanded in the presence of persistent TCR stimulation with anti-CD3 and anti-CD28 costimulation for 8 days. Different from A2AR^–/–^ CD8^+^ T cells, A2BR^–/–^ CD8^+^ T cells showed a greater ability to survive (Supplemental Figure 4, A and B), proliferate (Supplemental Figure 4C), and produce TNF-α (Supplemental Figure 4D) and IFN-γ (Supplemental Figure 4E) with less exhausted phenotype (Supplemental Figure 4, F and G) compared with WT CD8^+^ T cells upon chronic TCR stimulation. While adenosine, the endogenous agonist for A2AR and A2BR, was significantly upregulated in the metabolome of CD8^+^ T cells upon 4-1BB costimulation (Figure 1B), α4-1BB treatment decreased the levels of *a2br*, but not *a2ar*, transcripts in activated CD8^+^ T cells (Figure 5A), indicating a specific involvement of *a2br* signaling in 4-1BB–mediated CD8^+^ T cell responses. Indeed, intracellular GSH levels increased further in A2BR^–/–^ CD8^+^ T cells upon 4-1BB costimulation, validated by mBBr staining (Figure 5B and Supplemental Figure 4H) and a GSH/GSSG-Glo Assay Kit (Figure 5C). Consistently, there were higher expression levels of GCLC (Figure 5D) and GPX4 (Figure 5E) in A2BR^–/–^ CD8^+^ T cells than in WT T cells upon 4-1BB costimulation. The viability (Figure 5F) and proliferation (Figure 5G) were also improved, with comparable production of IFN-γ (Figure 5H) and TNF-α (Figure 5I) in A2BR^–/–^ CD8^+^ T cells upon 4-1BB costimulation. Interestingly, the frequency of exhausted-like PD-1^+^ (Figure 5J) and CD39^+^ cells (Figure 5K) was significantly decreased in A2BR^–/–^ CD8^+^ T cells compared with WT T cells upon 4-1BB costimulation. Earlier studies demonstrate that 4-1BB costimulation induces T cell mitochondrial function and biogenesis (15), which was supported by our RNA-Seq revealing one of the enriched pathways related to mitochondrion organization (Figure 1A). Furthermore, transmission electron microscopy showed that α4-1BB treatment expanded greater cristae folds in mitochondria, especially in A2BR^–/–^ CD8^+^ T cells, leading to more fission (Figure 5, L and M). More importantly, treatment of the GPX4 inhibitor RSL3 resulted in a loss of viability (Figure 5N) and GSH levels (Figure 5O) for activated CD8^+^ T cells even with A2BR ablation upon 4-1BB agonism. These data together suggest A2BR deletion helps sustain the GSH/GPX4 axis and α4-1BB–mediated CD8^+^ effector T cell survival and expansion while reducing their exhausted-like phenotype.

### A2BR deletion augments tumor-specific CD8^+^ T cell immunity and enables superior antitumor effects of ACT in combination with agonistic α4-1BB treatment.

**Figure 6:**
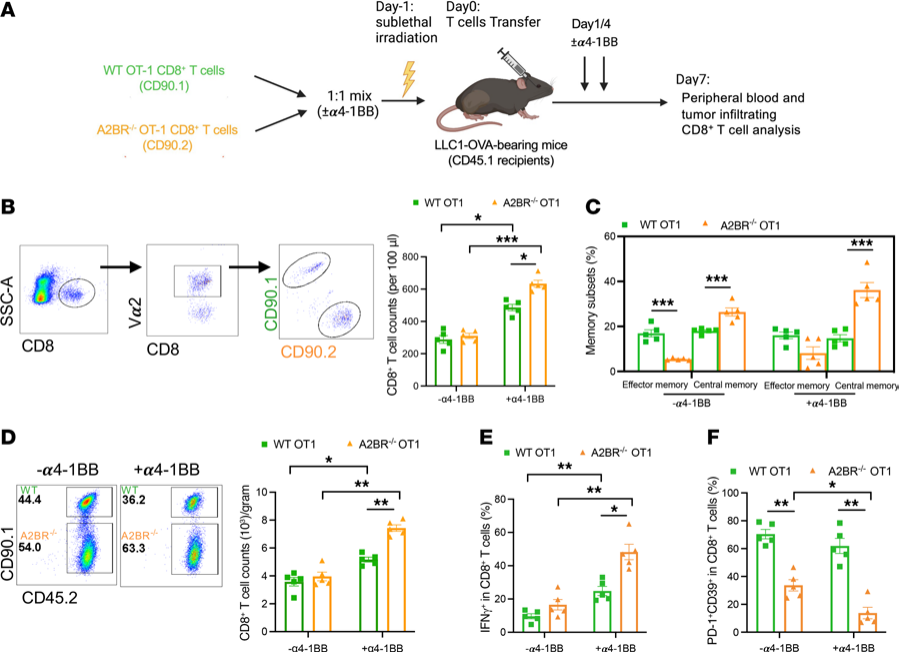
α4-1BB treatment preferentially promotes A2BR^–/–^ CD8^+^ T cell responses. For the T cell adoptive transfer in an LLC1-Ova tumor model (**A**), tumor-bearing CD45.1 mice (*n* = 5) were sublethally irradiated followed by i.v. injection of purified CD8^+^ T cells from CD90.1 OT-I WT or CD90.2 OT-I A2BR^–/–^ mice ex vivo stimulated by OVA-I peptide and anti-CD28 with or without anti-4-1BB (100 ng/mL). The group of mice with transfer of α4-1BB–treated T cells received additional doses of anti–4-1BB (100 μg/mouse) by i.p. daily once for 2 days as indicated. Donor-derived specific CD8^+^ T cells in peripheral blood and TILs were analyzed 7 days after transfer. (**B**) Relative count of OT-I WT or A2BR^–/–^ CD8^+^ T cells in transferred cells in peripheral blood. (**C**) Frequency of CD44^+^CD62L^–^ effector memory or CD44^+^CD62L^+^ central memory subsets in transferred T cells in peripheral blood. (**D**) Relative count of OT-I WT or A2BR^–/–^ CD8^+^ T cells in transferred cells in tumor infiltrates. IFN-γ^+^ cells (**E**) and PD-1^+^ CD39^+^ cells (**F**) among transferred tumor-infiltrating OT-I WT or CD45.2 OT-I A2BR^–/–^ CD8^+^ T cells were determined by flow cytometry. Data (means ± SEM) are representative of 2 (**B**–**F**) independent experiments. (**B**–**F**) Two-way ANOVA with Bonferroni’s post test correction was used. *P < 0.05; **P < 0.01; ***P < 0.001.

To determine how A2BR deletion affects tumor-specific CD8^+^ T cell responses in vivo upon α4-1BB treatment, preactivated OT-1 WT (CD90.1^+^) and A2BR^–/–^ (CD90.2^+^) CD8^+^ T cells congenic for CD45.2 were mixed at a 1:1 ratio and cotransferred into LLC1-Ova tumor–bearing recipients (CD45.1^+^) followed with or without α4-1BB treatment. Donor-derived specific CD8^+^ T cells in peripheral blood and TILs were analyzed 7 days after transfer (Figure 6A). Similar to what we observed in vitro, there was an increased accumulation of A2BR^–/–^ cells in transferred peripheral CD8^+^ T cells upon α4-1BB treatment (Figure 6B), and these A2BR^–/–^ cells showed more central memory phenotype (CD44^+^CD62L^+^) especially upon α4-1BB treatment compared with transferred OT1 WT T cells (Figure 6C). Likewise, α4-1BB treatment increased the numbers of A2BR^–/–^ cells in transferred CD8^+^ T cells within tumors (Figure 6D). Moreover, more tumor-infiltrating A2BR^–/–^ CD8^+^ T cells produced IFN-γ production (Figure 6E) with less exhausted-like state and high coinhibitory molecule expression of PD-1 and CD39 (35) (Figure 6F) compared with transferred WT CD8^+^ T cells.

**Figure 7:**
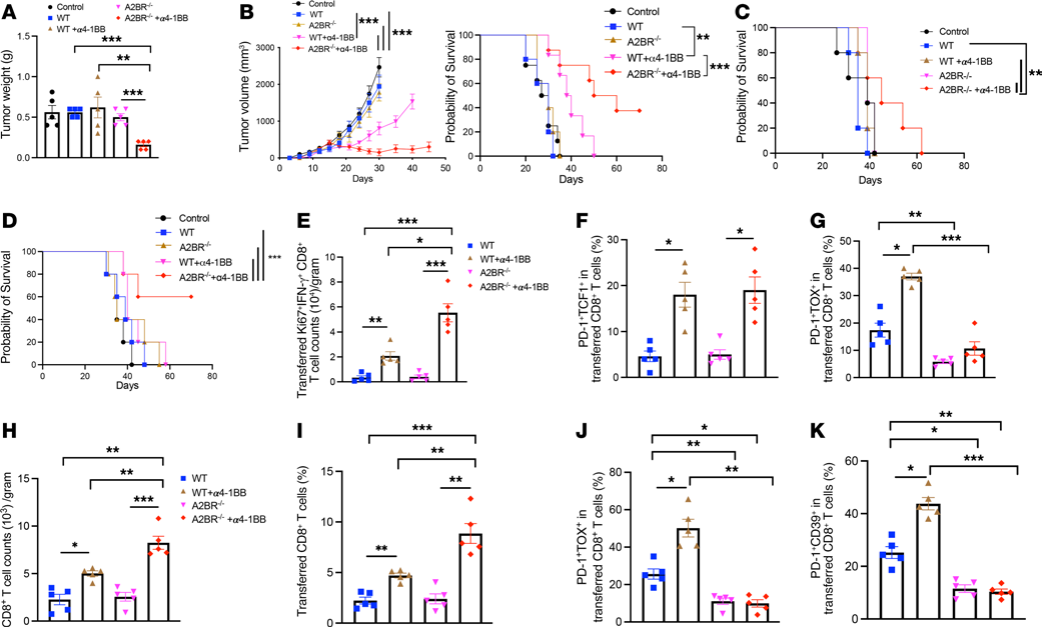
A2BR deletion sensitizes ACT to α4-1BB treatment. (**A**) LLC1-Ova tumor–bearing mice (*n* = 5) received sublethal irradiation followed by i.v. transfer of OT-I WT and A2BR^–/–^ CD8^+^ T cells treated ex vivo with αCD3/αCD28 with or without α4-1BB. The group of mice with transfer of α4-1BB–treated T cells received additional doses of α4-1BB treatment (100 μg/mouse) by i.p. daily once for 2 days. Tumors were harvested and weighed 22 days after T cell transfer. Similar experiments were conducted in E0771-SIY–bearing CD90.1 mice with transfer of 2C WT and A2BR^–/–^ CD8^+^ T cells and B16F10-bearing CD90.1 mice with transfer of Pmel WT and A2BR^–/–^ CD8^+^ T cells as well as in MC38-bearing CD90.1 mice with transfer of polyclonal tumor-reactive WT and A2BR^–/–^ CD8^+^ T cells. (**B**) Tumor growth and survival curves of E0771-SIY–bearing mice (*n* = 5–8) were plotted. Survival curves of B16F10-bearing mice (*n* = 5) (**C**) and MC38-bearing mice (*n* = 5) (**D**) were plotted. In parallel, E0771-SIY tumors (*n* = 5) treated as in **A** were harvested for immune characterization of Ki67^+^IFN-γ ^+^ CD8^+^ T cell counts (**E**), and frequency of TCF1^+^PD-1^+^ (**F**) or TOX^+^PD-1^+^ (**G**) cells in transferred tumor-infiltrating CD90.2^+^CD8^+^ T cells. Similarly, relative counts (**H**) and frequency (**I**) of transferred CD8^+^ T cells, frequency of exhausted-like subset TOX^+^PD-1^+^ (**J**) or CD39^+^PD-1^+^ cells (**K**) among transferred CD8^+^ T cell infiltrates were determined in B16F10-bearing mice treated as in **C**. The gating strategy is shown in Supplemental Figure 10. Data (means ± SEM) are representative of 2 (**A**, **B**, and **E**–**K**) or 3 (**C**) independent experiments. (**A**, **B**, and **E**–**K**) One-way ANOVA in combination with Dunnet’s test to correct for multiple comparisons and the log-rank test for survival curves (**B**–**D**) were used. *P < 0.05; **P < 0.01; ***P < 0.001.

We next tested the antitumor effects of ACT using tumor-specific A2BR^–/–^ versus WT CD8^+^ T cells in combination with α4-1BB. In an LLC1-Ova tumor model, ACT using either OT-1 A2BR^–/–^ versus WT CD8^+^ T cells failed to reduce tumor burden (Figure 7A). However, the antitumor effect was observed only in mice receiving α4-1BB plus adoptive transfer of OT-1 A2BR^–/–^ rather than WT T cells (Figure 7A). In an E0771-SIY tumor model, a significant increase in tumor inhibition and overall survival was observed in mice receiving α4-1BB plus ACT using SIY-specific 2C A2BR^–/–^ CD8^+^ T cells compared with that using 2C WT CD8^+^ T cells (Figure 7B). Similarly, only α4-1BB in combination with Pmel A2BR^–/–^ T cell therapy was able to increase survival of mice bearing B16F10 tumors (Figure 7C). Moreover, transfer of tumor-reactive polyclonal CD8^+^ T cells also enabled a therapeutic survival advantage in MC38 tumor–bearing mice with α4-1BB therapy (Figure 7D and Supplemental Figure 5). In the meantime, we found an increased infiltration of Ki67- and IFN-γ–expressing CD8^+^ T cells (Figure 7E), accompanied by a higher frequency of stem-like or progenitor-exhausted PD-1^+^TCF1^+^CD8^+^ T cells (Figure 7F) in transferred TILs from E0771-bearing mice receiving α4-1BB plus adoptive transfer of 2C A2BR^–/–^ T cells compared with WT T cells. Interestingly, α4-1BB treatment resulted in an increase in the frequency of both stem-like or progenitor-exhausted PD-1^+^TCF1^+^CD8^+^ T cells (Figure 7F) and PD-1^+^TOX^+^ exhausted CD8^+^ T cells (Figure 7G) in transferred 2C WT T cells, consistent with the findings of an earlier study (43). In contrast, α4-1BB treatment enhanced only the stem-like or progenitor-exhausted phenotype in transferred 2C A2BR^–/–^ T cells (Figure 7F), which exhibited a reduced exhausted state compared with transferred 2C WT T cells (Figure 7G). In B16F10 tumor–bearing mice, α4-1BB in combination with Pmel A2BR^–/–^ T cell therapy resulted in a significant increase in transferred CD8^+^ T cell accumulation (Figure 7, H and I) with a decreased exhausted phenotype (PD-1^+^TOX^+^ and PD-1^+^CD39^+^) (Figure 7, J and K) compared with transferred Pmel WT T cells. Considering that downstream of A2BR, the Gαs-PKA/ phosphorylated CREB (pCREB) signaling axis promotes CD8^+^ T cell dysfunction/exhaustion (44), we measured the pCREB levels of transferred T cells induced by α4-1BB treatment. Notably, tumor antigen–experienced effector PD-1^+^CD39^–^ and exhausted PD-1^+^CD39^–^ T cells expressed higher levels of pCREB than counterparts (Supplemental Figure 6A). Transferred Pmel A2BR^–/–^ T cells expressed lower levels of pCREB than transferred WT T cells under α4-1BB treatment (Supplemental Figure 6B), in line with their less exhausted phenotype (Figure 7, J and K). Similarly, there was an increased infiltration of transferred cells (Supplemental Figure 7A), accompanied by a lower frequency of exhausted PD-1^+^TOX^+^CD8^+^ T cells (Supplemental Figure 7B) in transferred TILs from LLC1-Ova tumor–bearing mice receiving α4-1BB plus adoptive transfer of OT-1 A2BR^–/–^ T cells compared with WT T cells. Taken together, these data suggest that A2BR ablation improves the persistence and effector function of transferred antitumor T cells with a less exhausted phenotype, enhancing the efficacy of α4-1BB therapy. In addition, we confirmed superior antitumor effects of α4-1BB therapy in tumor-bearing A2BR^–/–^ mice compared with WT mice without ACT (Supplemental Figure 8A), which correlated well with increased infiltration, proliferation, and IFN-γ production of endogenous tumor-infiltrating CD8^+^ T cells (Supplemental Figure 8, B–D).

## Discussion

Our findings underscore the importance of the GSH/GPX4 metabolic axis in the potentiation of antitumor CD8^+^ T cell responses by 4-1BB agonism (Supplemental Figure 9). We have shown that α4-1BB treatment results in upregulation of GCLC and GPX4, which enhances redox functional fitness, leading to increased T cell survival, expansion, and effector function. In T cells, GCLC, the rate-limiting enzyme subunit for GSH de novo synthesis, is transcriptionally induced after TCR activation. Mak et al. reported that *Gclc* ablation in T cells did not influence initial TCR signaling, but failed to reprogram the cellular machinery needed for clonal expansion (45). Similarly, antigen-specific T cells lacking *Gpx4* failed to expand and to protect from acute infections while being susceptible to ferroptosis because of accumulation of membrane lipid peroxides (39). Subsequent studies also indicate that GPX4 protects follicular helper T cells from ferroptosis (46). By treating the activated CD8^+^ T cells with BSO or Prima to reduce intracellular GSH levels, we have obtained evidence that GSH metabolism is required to support the proliferation and IFN-γ production of the αCD3/CD28-activated CD8^+^ T cells as well as the enhanced proliferation and IFN-γ production of α4-1BB–treated CD8^+^ T cells. Furthermore, the ability of α4-1BB treatment to promote proliferation and IFN-γ production in vitro and in vivo is abrogated in antigen-specific GPX4^–/–^ CD8^+^ T cells compared with WT T cells from tumor-bearing mice. Either genetic alation or pharmacological inhibition of GPX4 also results in substantial ferroptosis of anti–4-1BB–treated CD8^+^ T cells. On the other hand, several recent studies reported a weakened antitumor function of tumor-infiltrating CD8^+^ T cells through increased lipid peroxidation and concomitant ferroptosis (47–49). These results together indicate that 4-1BB costimulation facilitates at least 3 fundamental properties of antitumor CD8^+^ T cells (survival, expansion, and effector function) metabolically through the GSH/GPX4 axis. The apparent increases in GSH synthesis and GPX4 activity, indicative of metabolic “rewiring” with improved mitochondrial function, are likely prepared to meet heightened energy and nutrient demands of activated CD8^+^ T cells for maintaining their survival, expansion, and effector functions, such as cytokine production during continuous antigenic activation, especially with 4-1BB costimulation. Indeed, we performed the [U-^13^C] glutamine isotope tracer assay to confirm the enhanced GSH synthesis and utilization together with more effective fueling of the TCA cycle by α4-1BB treatment. Our data support a critical role of the GSH/GPX4 axis for 4-1BB costimulation in metabolic reprogramming, complementing the recent findings of the key involvement of glucose and fatty acid metabolism (14) as well as mitochondrial quality and capacity (15) in 4-1BB costimulation. Nevertheless, the link between the GSH/GPX4 axis and other essential metabolic and mitochondrial changes during 4-1BB costimulation remains to be further determined.

We have identified the importance of the NF-κB signaling pathway for 4-1BB–mediated immunostimulatory activity on CD8^+^ T cells through activation of the GSH/GPX4 cascade. Specifically, both NF-κB1 and NF-κB2, the central components of the canonical and noncanonical NF-κB pathways, can bind to the *gpx4* promotor and dictate the induction of *gpx4* transcription upon 4-1BB costimulation. Remarkably, NF-κB inhibition–mediated immunosuppressive effects on T cells are recovered completely by the addition of cell-permeable GSH, highlighting the essential role of GSH metabolism in sustaining activation of the NF-κB downstream pathway responsible for 4-1BB agonism against oxidative stress. The PI3K/AKT, stress-activated protein kinase/JNK, p38 MAPK, and ERK1/2 pathways in addition to NF-κB pathways have been involved in 4-1BB–mediated T cell stimulation. Further studies in this important area will thus help us understand how interactions between the NF-κB pathway and the GSH/GPX4 cascade are integrated into other cellular signaling pathways upon 4-1BB costimulation.

We have previously shown that CD73-mediated adenosinergic action impedes the antitumor T cell immunity upon 4-1BB stimulation (22). We further demonstrated the role of A2AR signaling and the GSH/GPX4 axis in orchestrating metabolic fitness and survival of functionally competent CD8^+^ T cells (24). In the current study, however, we found that agonistic α4-1BB treatment results in decreased expression of A2BR rather than A2AR in activated CD8^+^ T cells. Furthermore, A2BR^–/–^ CD8^+^ T cells showed higher expression levels of GCLC and GPX4 than WT T cells upon 4-1BB costimulation, leading to greater levels of viability, proliferation, and IFN-γ production in vitro and in vivo. These results suggest that deletion of A2BR in the CD73-mediated adenosinergic cascade could sensitize antitumor CD8^+^ T cells to agonistic anti–4-1BB treatment durably through enhancement of the GSH/GPX4 metabolic axis. While the GPX4/GSH levels are correlated preferentially with T cell stemness (24), the specific role of GSH/GPX4 in T cell differentiation and exhaustion within the tumor requires further investigation, particularly in the context of 4-1BB costimulation. Notably, targeting the A2BR pathway seems sufficient to mitigate effector CD8^+^ T cell exhaustion (as determined by reduced expression of PD-1, TOX, and CD39 within tumors), linking to enhanced levels of GSH/GPX4, which is supported by an elegant study showing the importance of CD39 and adenosine signaling for suppressive function of tumor-infiltrating exhausted T cells in the hypoxia tumor microenvironment (50). Consistently, activation of the downstream A2AR or A2BR pathway, specifically the Gαs/PKA/pCREB signaling axis, has been shown to promote T cell exhaustion and immunotherapy failure (44). In addition, 4-1BB agonism can cause unwanted systemic expansion of exhausted T cells with limited effector functions involving activation of the NF-κB signaling pathway (43), which may account for the so-far inadequate efficacy of 4-1BB agonists. Taking these data together, we propose a more effective strategy in which agonistic anti–4-1BB in combination with A2BR deletion augments the survival and expansion of antitumor effector CD8^+^ T cells while protecting them from terminal exhaustion (Supplemental Figure 9). This improvement to CD8^+^ TIL effector functionality and persistence via A2BR deletion upon α4-1BB treatment leads to better control of tumor progression and enhanced therapeutic efficacy. On the other hand, TILs express variable amounts of A2AR in addition to A2BR, accounting for the adenosine-mediated inhibitory effect on T cell metabolic fitness (23). Along with the evidence of A2AR and A2BR expression in tumor cells and different immune cell populations, further investigations are thus needed to explore whether inactivation of A2BR and/or A2AR in both tumor and immune cellular compartments elicits optimal antitumor T cell immunity upon 4-1BB costimulation by modulation of T cell metabolic fitness.

## Methods

### Sex as a biological variable.

Both male and female animals were examined in this study, and similar findings were reported for both sexes.

### Mice, cell lines, and reagents.

C57BL/6J WT, Pmel, OT-I, Gpx4^fl/fl^, CD4-cre, CD90.1, CD45.1, and C57BL/6 SCID mice were purchased from the Jackson Laboratory. The A2BR^–/–^ mice have been described previously (51). Hans Schreiber (University of Chicago, Chicago, Illinois, USA) provided B16F10 cell lines. The MC38 cell line was obtained from Arlene Sharpe (Harvard Medical School, Boston, Massachusetts, USA). LLC1 cells expressing OVA (LLC1-Ova) and E0771-SIY were generated as previously described (25). All these cell lines were routinely tested for mycoplasma infections by culture and DNA stain and maintained in complete medium composed of RPMI 1640 with 5% FBS. The GSH/GSSG-Glo Assay Kit was purchased from Promega. Anti-mouse 4-1BB (3H3 and LOB12.3) antibodies were purchased from Bio X Cell. Cell-permeable GSH (glutathione ethyl ester) and Prima-1^met^ were purchased from Cayman Chemical Company. Bay 11-7082, liproxstatin-1, and RSL-3 (1S,3R–) were purchased from MedChem Express. BSO was purchased from Sigma-Aldrich. mBBr was purchased from Invitrogen. BODIPY 581/591 C11 (BODIPY), MitoTracker Green FM, and MitoTracker Red CMXRos were purchased from Thermo Fisher Scientific. [U-^13^C] glucose and [U-^13^C] glutamine were purchased from Cambridge Isotope Laboratories. Antibodies for functional studies are anti-mouse CD3 (clone 145-2C11, BioLegend, catalog 100360, working concentration: 1 μg/mL) and anti-mouse CD28 (clone 37.51, BioLegend, catalog 102121, working concentration: 1 μg/mL). Antibodies for flow cytometry are anti-mouse CD8 (53-6.7, BioLegend, catalog 100712 and 100725, BD Biosciences, catalog 612759), anti-mouse IFN-γ (XMG1.2, BioLegend, catalog 505826, 505808 and 505842), anti-mouse TNF-α (MP6-XT22, BioLegend, catalog 506333), anti-mouse Ki67 (11F6 and 16A8, BioLegend, catalog 151212 and 652404), anti–annexin V (BioLegend, catalog 640941), 7-AAD (BioLegend, catalog 420404), anti-mouse CD90.1 (OX-7, BioLegend, catalog 202516,HIS51, Invitrogen, catalog 25-0900-82), anti-mouse CD45.1 (BioLegend, catalog 110706 and Invitrogen, catalog 45-0453-82), anti-mouse CD45.2 (BioLegend, catalog 109820), anti-mouse Vα2 (B20.1, BioLegend, catalog 127810 and 127808), anti-mouse PD-1(29F.1A12, BioLegend, catalog 135214), anti-mouse CD39 (BioLegend, catalog 143806), anti-CREB (pS133)/ATF-1 (pS63) (clone J151-21, BD, catalog 558434), anti-mouse GPX4 (EPNCIR144, Abcam, catalog ab125066), GCLC (EPR20078, Abcam, catalog ab207777) and p-IKKαβ (Ser176180) (16A6) rabbit mAb (Alexa Fluor 647 conjugate, Cell Signaling Technology, catalog 36516). Antibodies for ChIP are RNA pol II (MilliporeSigma, catalog 05-623), normal mouse IgG (MilliporeSigma, catalog 12-171), NF-κB1 antibody (p65, Thermo Scientific, catalog 14-6731-81), and NF-κB2 antibody (p100, Thermo Fisher Scientific, catalog 10409-2-AP).

### Analysis of cells by flow cytometry.

All samples were initially incubated with 2.4G2 to block antibody binding to Fc receptors. Single-cell suspensions were stained with 1 μg of relevant mAbs and then washed twice with cold PBS. For cell-apoptosis analysis, cells were rinsed with 1× binding buffer, and then stained with annexin V and 7-AAD along with other surface antibodies in binding buffer at room temperature (RT) in the dark for 10 minutes and immediately run on a flow cytometer. Ki67 nuclear staining was performed according to the manufacturer’s instructions (eBioscience). Intracellular IFN-γ and TNF-α staining under stimulation with 50 ng/ml PMA, 5 μg/ml ionomycin plus 10 μg/ml BFA in the presence of the relevant tumor antigen peptides was performed according to the manufacturer’s instructions (BD Bioscience). For phosphoprotein staining, cells were fixed and permeabilized using the Phosflow Kit (BD Bioscience) according to the manufacturer’s protocol before staining for 1 hour at room temperature. For BODIPY and mBBr staining, cells were washed with 2 ml of PBS and centrifuged at 300*g* for 5 minutes at RT. The cells were then resuspended in 0.5 ml of PBS prewarmed to 37°C. BODIPY was added at the concentration of 5 μM and gently vortexed. The cell suspension was incubated with BODIPY for 20 minutes at 37°C. In parallel, the mBBr was added at a concentration of 50 μM, and the cell suspension was incubated for 10 minutes at 37°C. The cells were washed with 1 ml of PBS and centrifuged at 300*g* for 5 minutes at 4°C prior to flow cytometric detection. For measurement of mitochondrial membrane potential and mass, CD8^+^ T cells were stained with 200 nM MitoTracker Red CMXRos (MTR) and 100 nM MitoTracker Green FM (MTG) for 15 minutes at 37°C. Samples were harvested on LSRII and FACSCanto, and data were analyzed with FlowJo software.

### RNA extraction and real-time PCR.

Total RNA was extracted using TRIzol reagent (Invitrogen) according to the manufacturer’s instructions. The cDNA synthesis was performed using SuperScript One-Step RT-PCR (Invitrogen). Quantitative real-time PCR was used to quantify genes by SYBR Green (Bio-Rad), and relative abundance of each mRNA was normalized to β-actin mRNA. The primers used for q-RT-PCR are as follows: β-actin: F: 5′-TAGGGATGTCAACGTCACAC-3′, R: 5′-AAGAGCTATGAGCTGCCTGA-3′; GPX4: F: 5′-GCAACCAGTTTGGGAGGCAGGAG-3′, R: 5′-CCTCCATGGGACCATAGCGCTTC-3′; GCLC: F: 5′-AGAACACGGGAGGAGAGAGG-3′, R: 5′-CTTACTGATCCTAAAGCGATTGTTC-3′; NF-κB1: F: 5′-GCTGCCAAAGAAGGACACGACA-3′, R: 5′-GGCAGGCTATTGCTCATCACAG-3′; NF-κB2: F: 5′-TGCTGATGGCACAGGACGAGAA-3′, R: 5′-GTTGATGACGCCGAGGTACTGA-3′.

### In vitro 4-1BB costimulation of CD8^+^ T cells.

CD8^+^ T cells were purified using EasySep Mouse CD8a Selection Kits (STEMCELL Technologies Inc.) from WT, A2BR^–/–^, GPX4^fl/fl^ CD4-cre, OT1, Pmel, OT-I A2BR^–/–^, or Pmel A2BR^–/–^ mice. These T cells were subsequently stimulated with anti-CD3 (1 μg/ml), peptide OVA-I (0.1 μg/ml), gp100 (0.5 μg/ml), or anti-CD28 (1 μg/ml) in the presence or absence of anti-mouse 4-1BB (clone 3H3 or LOB12.3, 100 ng–500 ng/ml). On day 4, cells were counted and analyzed for T cell apoptosis, cytokine production, and proliferation by flow cytometry.

To investigate the effects of GSH in NF-κB signaling in CD8^+^ T cell effector functions, Bay 11-7082 (3.5 μM) was added with/without GSH (500 μM) in CD8^+^ T cells culture on day 0 with TCR stimulation as described above. On day 1, cells were counted and analyzed for T cell apoptosis, cytokine production, and proliferation by flow cytometry.

### Tumor challenge and adoptive T cell therapy.

1 × 10^6^ cells of B16F10, E0771-SIY, LLC1-Ova, or MC38 in suspension were injected s.c. into the lower right flank of mice on day 0. For the T cell adoptive transfer experiments, tumor-bearing mice were sublethally irradiated with 600 cGy on day 7. Purified CD8^+^ T cells from Pmel, 2C, OT-1, or nontransgenic WT mice were cultured in the presence of anti-CD3 or antigen peptides and anti-CD28 with/without anti–4-1BB (100 ng/mL) for 24 hours. On day 8, activated CD8^+^ T cells were transferred i.v. at 1 × 10^6^ per mouse. MC38 tumor-reactive effector T cells were prepared as described previously (24). Tumor volumes were measured along 3 orthogonal axes (a, b, and c) and calculated as abc/2 every 2–4 days. Tumor volume beyond 1500 cm^3^ was regarded as end point, which was recorded for survival curve.

### Adoptive T cell transfer and immune characterization in tumor-bearing mice.

LLC1-Ova cells (1 × 10^6^ per mouse) in suspension were injected s.c. into the lower right flank of CD90.1 host mice on day 0. For the T cell adoptive transfer experiments, tumor-bearing mice were sublethally irradiated with 600 cGy when the tumor sizes were about 250 mm^3^. Purified CD8^+^ T cells from CD45.1 OT-1 WT or CD45.2 OT-1 GPX4^–/–^ mice were cultured in the presence of anti-CD3, OVA-I peptide, or anti-CD28 with/without anti–4-1BB (100 ng/mL) for 24 hours. These activated WT and A2BR^–/–^ CD8^+^ T cells were then mixed at a ratio of 1:1 and transferred i.v. at 2 × 10^6^ per mouse. The group of mice with adoptive transfer of ant–4-1BB–treated T cells received additional doses of anti–4-1BB treatment (100 μg/mouse) by i.p. daily once for 2 days. Peripheral blood was drawn by retroorbital bleeding and tumor-infiltrating cells were analyzed 5–8 days after T cell transfer. Similar experiments were performed using CD45.1 recipient mice injected with LLC1-Ova cells. For adoptive T cell transfer, purified CD8^+^ T cells from CD90.1 OT-1 WT or CD90.2 OT-1 A2BR^–/–^ mice were treated with or without anti-4-1BB as above.

### Electron microscopy for mitochondria morphology.

CD8^+^ T cells were stimulated by anti-CD3 and anti-CD28 with or without anti–4-1BB (100 ng/mL) for 4 days. Cells were then washed with PBS and gently resuspended in fixative buffer (3% glutaraldehyde, 2% formaldehyde in 0.1 M PIPES, pH 7.2) for 1 hour at RT. Samples were analyzed in Northwestern University NUANCE’s BioCryo facility. The number of mitochondria per cell was counted. For mitochondrial cristae, the crista number or total length in one mitochondrion was calculated from high-magnitude electron microscopy (EM) images of live cells.

### LC-MS metabolite analysis.

For in vitro T cell tracing experiments, Pmel or OT-I CD8^+^ T cells were stimulated by anti-CD3 and anti-CD28 with or without anti–4-1BB (100 ng/mL) for 4 days. These activated T cells were then counted in duplicate and resuspended in glutamine-free growth RPMI media with [^13^C-U]-glutamine (4 mM) or glucose-free growth RPMI media with [^13^C-U]-glucose (11 mM) and incubated for 4 hours at 37°C at 2 × 10^6^ viable cells per mL. For both metabolomics and tracing experiments, treated CD8^+^ T cells were collected and washed with cold saline solution twice in 15 ml tubes. After centrifugation at 300*g* for 5 minutes at 4°C, 1 mL of 80% cold methanol was added. The cell lysate/methanol mixture was transferred to a 1.5 mL conical tube and frozen in liquid nitrogen. This was subjected to 3 freeze-thaw cycles between liquid nitrogen and 37°C, vortex 30” after each thaw. The samples were again centrifuged at 20,000*g* for 15 minutes at 4°C. The supernatant was collected in new tubes and normalized by cell number. Samples were kept at –80°C until measured. The detection of intracellular metabolites in CD8^+^ T was performed at the Northwestern University Metabolomics Core Facility using LC-MS.

### ChIP assay.

ChIP assay was performed with the ChIP IT-EXPRESS Kit following the manufacturer’s protocol (Active Motif, catalog 53008). Activated CD8^+^ T cells treated with or without anti-mouse 4-1BB were fixed, lysed and, sonicated to prepare chromatin samples. Immunoprecipitation was performed with positive control antibody (RNA pol II, MilliporeSigma, catalog 05-623), negative control antibody (normal mouse IgG, MilliporeSigma, catalog 12-171), NF-κB1 antibody (p65, Thermo Fisher Scientific, catalog 14-6731-81), and NF-κB2 antibody (p100, Thermo Fisher Scientific, catalog 10409-2-AP). ChIP-enriched chromatin was used for real-time PCR. Relative expression levels were normalized to input. The primers used for ChIP assay are as follows: GADPH F: 5′-GCCCTTCTACAATGCCAAAG-3′, R: 5′-TTGTGATGGGTGTGAACCAC-3′; GPX4#1 F: 5′-AGCTGGTCCTGTCAGTTGTT-3′, R: 5′-CCCGGCCTAACAGTGGAAG-3′; GPX4#2 F: 5′-TATCCCGGCTTGTGTCCTCC-3′, and R: 5′-GGGACAGTTCCTTTTCCTGGT-3′.

### Bulk RNA-Seq.

RNA from activated CD8^+^ T cells with or without anti-mouse 4-1BB was extracted using the Direct-zol RNA Miniprep Kit (Zymo Research) as described by the manufacturer’s protocol. The RNA of each sample was quantified using the Qubit RNA HS Assay Kit (Thermo Fisher Scientific), and RNA integrity was evaluated by the 2100 Bioanalyzer (Agilent Technologies). mRNA was isolated using the NEBNext Poly(A) mRNA Magnetic Isolation Module (New England Biolabs), and cDNA libraries were generated using the low-input strand-specific RNA-Seq kits NEBNext Ultra II RNA Library Prep Kit for Illumina (96 reactions) and NEBNext Multiplex Oligos for Illumina (96 index primers) (New England Biolabs). All RNA and DNA purification steps were performed using AMPure XP Beads (Beckman Coulter Inc.). The quantity and quality of cDNA were assessed using the Qubit dsDNA HS Assay Kit (Thermo Fisher Scientific). Libraries were sequenced at Northwestern University Sequencing Core (NUSeq) in the Illumina HiSeq 4000 platform using single-end 50 bp reads and generating an average 20 million reads per sample.

### Statistics.

Mean values were compared using an unpaired Student’s 1-tailed or 2-tailed *t* test as well as 1-way ANOVA analysis, unless indicated otherwise. The statistical differences in tumor growth between groups of mice were determined by 2-way ANOVA analysis. The statistical differences between the survival of groups of mice were calculated according to the log-rank test. The correlations between 2 elements were calculated by Pearson’s correlation. Probability values > 0.05 were considered nonsignificant.

### Study approval.

All animal experiments were approved by Northwestern University’s IACUC (IS00002155).

### Data availability.

Bulk RNA-Seq data have been deposited to the NCBI’s Gene Expression Omnibus database (GEO GSE222286). Values for data points shown in graphs and values behind means are reported in the Supporting Data Values file.

## Author contributions

JA, PX, and BZ conceived and designed the study. JA, PX, GS, SC, JF, MZ, and HT developed methodology. JA, PX, GS, SC, MZ, JF, HT, and ARZ acquired data (provided animals, acquired and managed patients, provided facilities, etc.). JA, PX, GS, SC, TMK, YZ, and BZ analyzed and interpreted data (e.g., statistical analysis, biostatistics, computational analysis). JA, SC, YW, YZ, DF, TMK, and BZ wrote, reviewed, and/or revised the manuscript. JA, PX, GS, SC, JF, and BZ provided administrative, technical, or material support (i.e., reporting or organizing data, constructing databases). BZ supervised the study.

## Supplementary Material

Supplemental data

Supporting data values

## Version 1 04/03/2025

In-Press Preview

## Version 2 06/02/2025

Electronic publication
