# Supplementary material for: 4-1BB stimulation with concomitant inactivation of adenosine A2B receptors enhances CD8+ T cell antitumor response
Source: J Clin Invest. 2025 Apr 3;135(11):e190841. doi: 10.1172/JCI190841 (PMC12126218; doi:10.1172/JCI190841)
Supplement: Supplemental data [file jci-135-190841-s144.pdf]

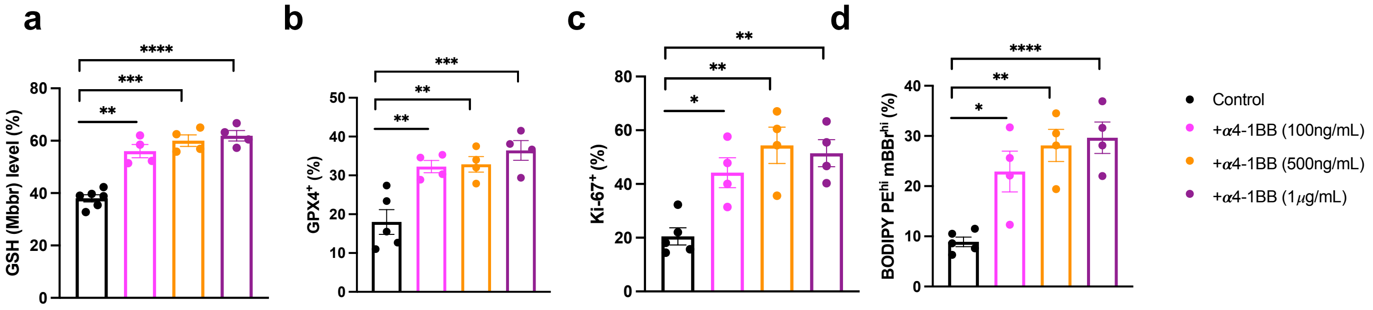

Figure S1. α4-1BB clone 3H3 increases CD8+ T cell functions effectively. CD8+ T cells were stimulated by anti-CD3 and anti-CD28 with or without α4-1BB (clone 3H3, 100, 500, or 1000 ng/ml) for 4 days. Expression levels of GSH (mBBr) (**a**), GPX4 (**b**), Ki-67 (**c**), and BODIPY-PE^{hi}mBBr^{hi} cells+ (**d**) were analyzed by flow cytometry. Data (mean ± SEM) are representative of 2 independent experiments. One-way ANOVA in combination with Dunnet's test to correct for multiple comparisons was used. *$p < 0.05$, **$p < 0.01$, ***$p < 0.001$; ****$p < 0.0001$.

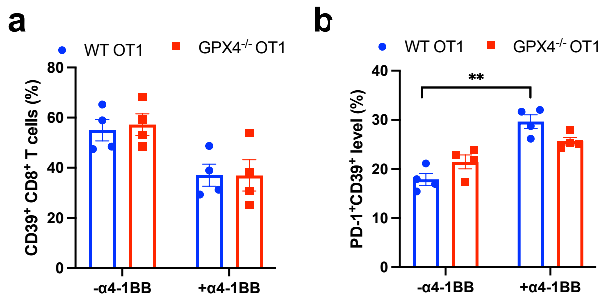

Figure S2. Under the setting of Fig. 3q, frequencies of CD39+CD8+ cells (**a**), and PD-1+CD39+CD8+ cells (**s**) among transferred tumor-infiltrating OT-I WT or CD45.2 OT-I GPX4-/- T cells were determined by flow cytometry.

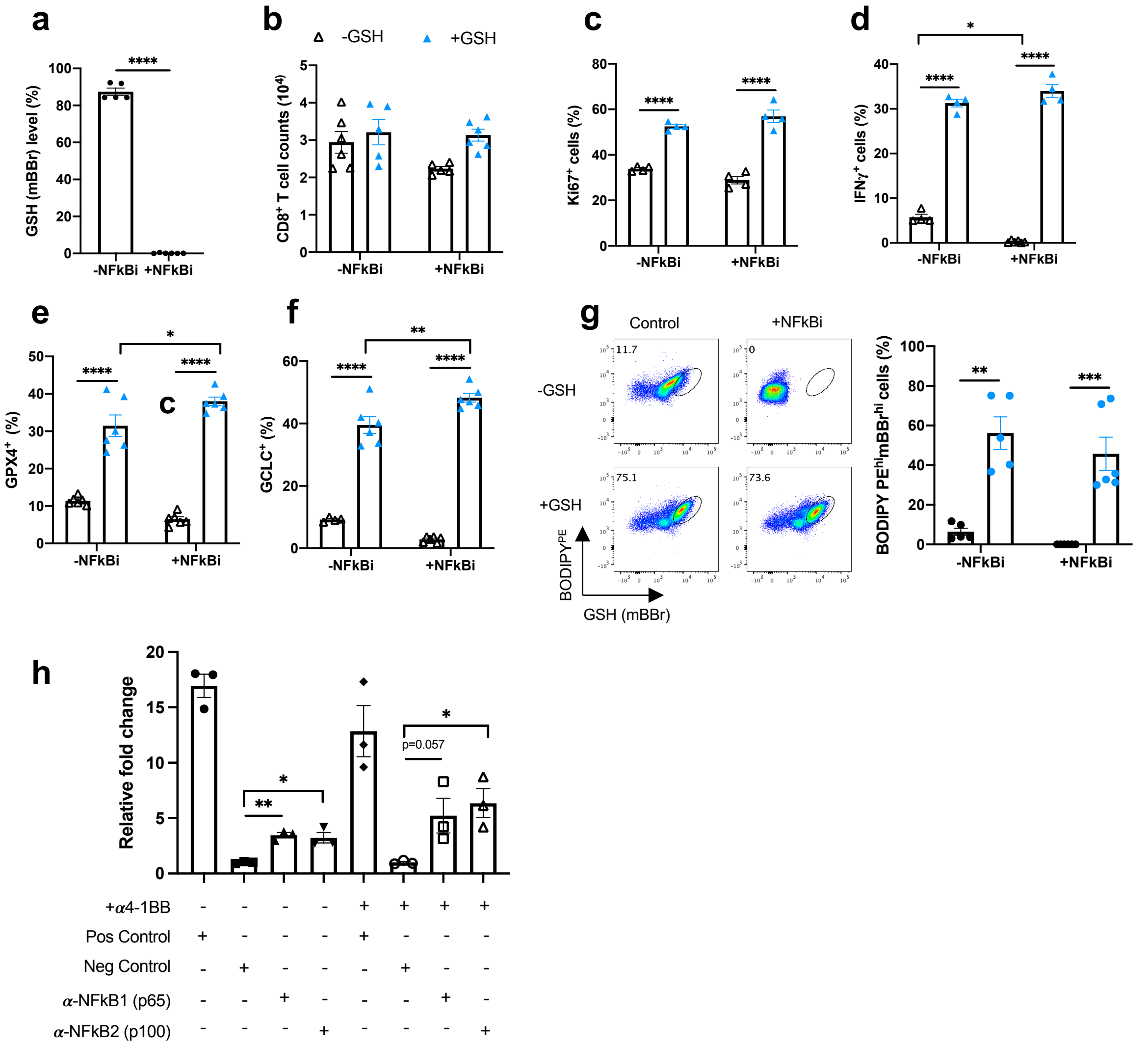

Figure S3. The NF-kB inhibitors (Bay 11-7082) and/or cell permeable GSH was added to CD8+ T cell cultures stimulated by anti-CD3 and anti-CD28 but without α4-1BB treatment. Intracellular GSH level (**a**), viable cell counts (**b**), Ki-67+ cells (**c**), IFN-γ+ cells (**d**), GCLC+ cells (**e**), GPX4+ cells (**f**), and BODIPY-PE^{hi}mBBr^{hi} cells (**g**) were analyzed by flow cytometry. (**h**) Chip-qPCR analysis confirming the enhanced binding capacity of NF-kb1 and NF-kb2 on *gpx4* (predicted binding site 2) in α4-1BB-treated CD8+ T cells compared to control cells. Data (mean ± SEM) are representative of 2 (**a-g**) independent experiments. (**a**) Unpaired Student's t test. (**b-h**) A two-way ANOVA with Bonferroni post-test correction was used. *$p < 0.05$, **$p < 0.01$, ***$p < 0.001$; ****$p < 0.0001$.

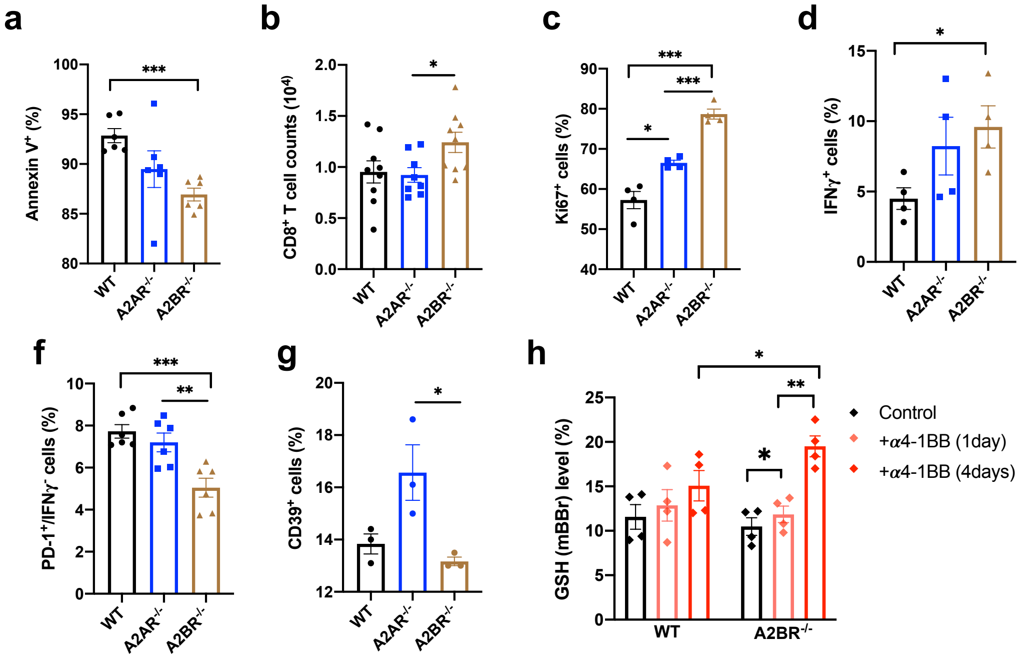

Figure S4. A2BR deletion promotes T cell function in continued TCR stimulation. WT, A2AR$^{-/-}$, and A2BR$^{-/-}$ CD8^{+} T cells were activated with αCD3/28 for 8 days (half of the medium with αCD3/28 replaced every 2 days). The Annexin V^{+} cells (**a**), viable cell counts (**b**), Ki-67^{+} cells (**c**), IFN-γ^{+} cells (**d**), TNF-α^{+} cells (**e**), PD-1^{+}IFN-γ^{-} cells (**f**), and CD39^{+} cells (**g**) were determined by flow cytometry. WT and A2BR$^{-/-}$ CD8^{+} T cells were stimulated by anti-CD3 and anti-CD28 with or without α4-1BB (clone 3H3, 100, 500, or 1000 ng/ml) for 1 day or 4 days. Expression levels of GSH (mBBr) (**h**) were analyzed by flow cytometry. Data (mean ± SEM) are representative of 3 (**a-g**) and 2 (**h**) independent experiments. One-way ANOVA in combination with Dunnet's test to correct for multiple comparisons was used. *$p < 0.05$, **$p < 0.01$, ***$p < 0.001$.

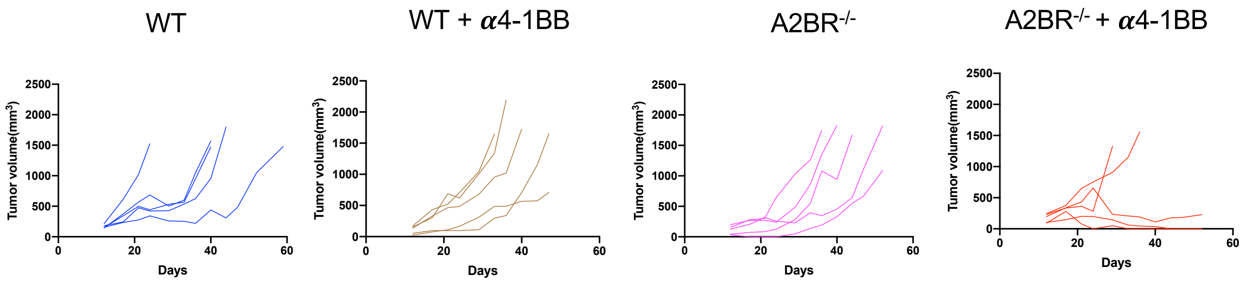

Figure S5. Adoptive T cells therapy with A2BR$^{-/-}$ CD8^{+} T cells in combination with α4-1BB treatment. One of representative experiment involving MC38 tumor-bearing mice (n=5) received sublethal irradiation followed by i.v. transfer of polyclonal WT and A2BR$^{-/-}$ CD8^{+} T cells treated *ex vivo* with αCD3/αCD28 with or without α4-1BB. The group of mice with transfer of α4-1BB-treated T cells received additional doses of α4-1BB treatment (100 μg/mouse) by i.p. daily once for 2 days. Tumor curves of individual tumor-bearing mice were plotted**.**

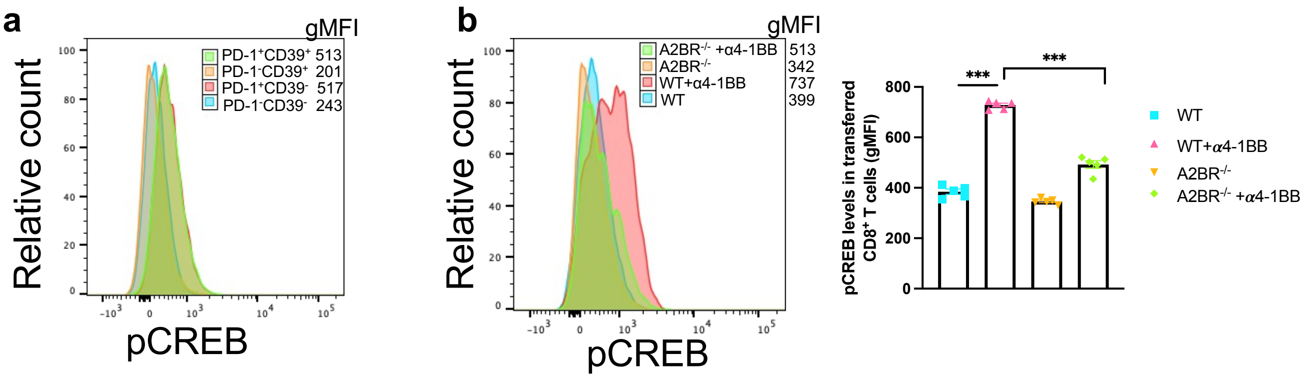

Figure S6. B16F10 tumors (n=5) treated as Fig. 7c were harvested on d7 for measurement of pCREB induction (determined by geometric mean fluorescence intensity, gMFI) in transferred Pmel T cells within the tumor by flow cytometry. (**a**) The expression levels of pCREB in indicated subsets among transferred infiltrating Pmel WT T cells without α4-BB treatment. (**b**) The expression levels of pCREB in transferred infiltrating Pmel WT or A2BR$^{-/-}$ T cells from tumor-bearing mice with α4-1BB or control IgG treatment. ***$p < 0.001$, One-way ANOVA in combination with Dunnet's test to correct for multiple comparisons was used. Data (mean ± SEM) are representative of 2 independent experiments.

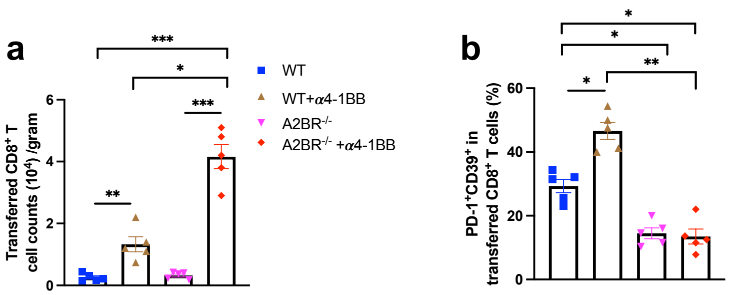

Figure S7. LLC1-Ova tumors (n=5) treated as Fig. 7**a** were harvested for immune characterization of transferred OT-1 T cell count ($CD8^+CD90.2^+CD3^+$) (**a**) and the frequency of exhausted-like cells ($CD8^+CD39^+PD\text{-}1^+$) (**b**) among those transferred OT-1 $CD8^+$ tumor infiltrates determined by flow cytometry. *$p < 0.05$, **$p < 0.01$, One-way ANOVA in combination with Dunnet's test to correct for multiple comparisons was used. Data (mean ± SEM) are representative of 2 independent experiments.

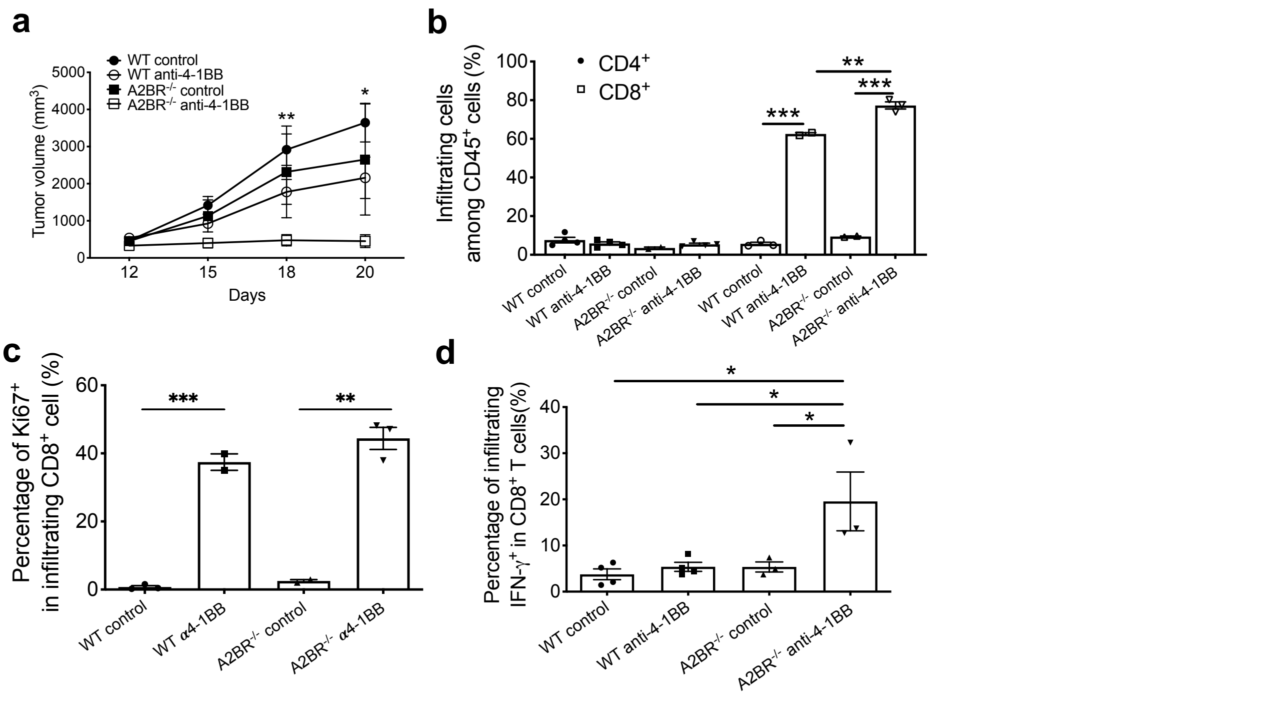

Figure S8. Host A2BR deletion promotes antitumor responses of α4-1BB treatment. B16-SIY tumor bearing WT and A2BR$^{-/-}$ mice (5 per group) were treated with or without α4-1BB (100 μg/mouse). Tumor growth curve was plotted (**a**) and tumor infiltrating CD4^{+} and CD8^{+} T cells (**b**) were analyzed on day 20. Percentages of Ki-67^{+} cells (**c**) and IFN-γ^{+} cells (**d**) in tumor infiltrating CD8^{+} T cells were determined by flow cytometry. *$p < 0.05$, **$p < 0.01$, ***$p < 0.001$. A two-way ANOVA with Bonferroni post-test correction was used.

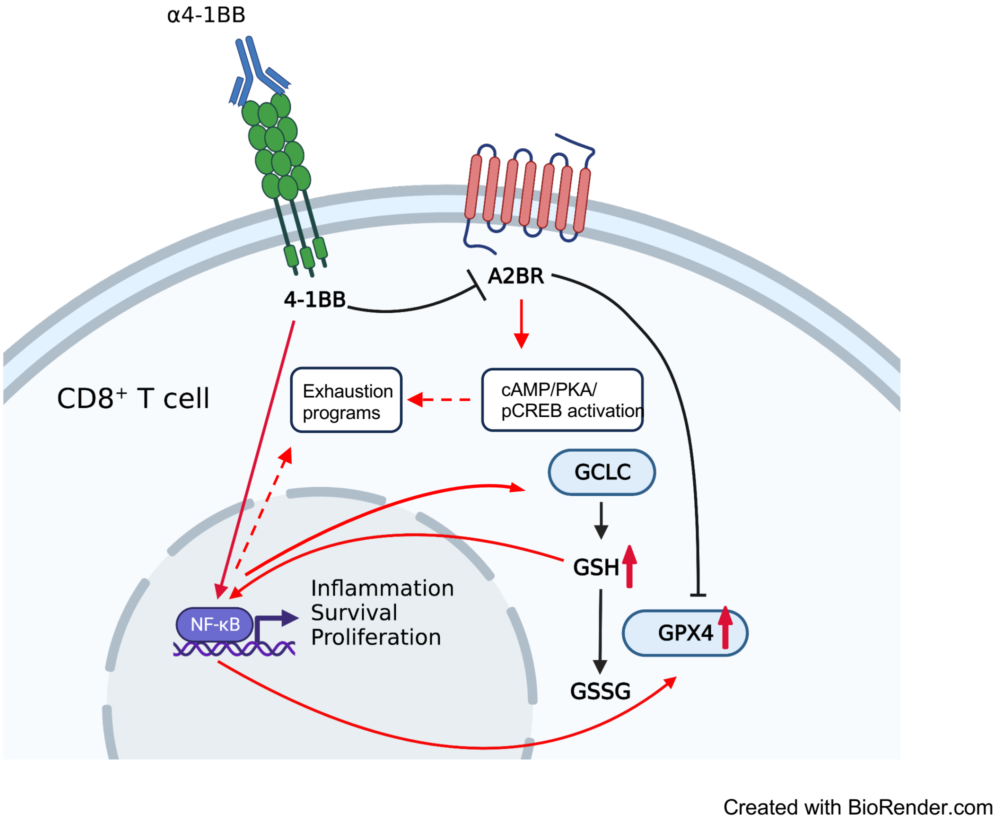

Figure S9. The proposed mechanism underlying α4-1BB-mediated enhancement of CD8⁺ T cell function involves modulating the GSH-GPX4 metabolic axis and A2BR signaling. Specifically, 4-1BB agonism upregulates GCLC and GPX4, which requires activation of the downstream NF-kB signaling pathway. This pathway sustains GSH metabolism and enhances redox functional fitness, leading to increased CD8⁺ T cell survival, expansion, and effector function. Furthermore, 4-1BB costimulation downregulates A2BR expression, and A2BR deletion potentiates the GSH-GPX4 metabolic cascade, improving CD8⁺ T cell persistence and function while reducing their exhausted-like phenotype upon 4-1BB agonism. Given the potential role of cAMP-PKA-pCREB signaling and the NF-kB signaling pathway in promoting T cell exhaustion, targeting A2BR enhances the expansion of tumor-specific CD8⁺ T cells, protects them from an exhausted-like state, and leads to superior antitumor effects in combination with agonistic 4-1BB treatment during adoptive cell transfer (ACT). The red solid lines indicate inducing/promoting effects demonstrated here, while the red dashed lines represent resulting actions supported by the published results (43, 44) and correlated data presented here.

Gating Strategy

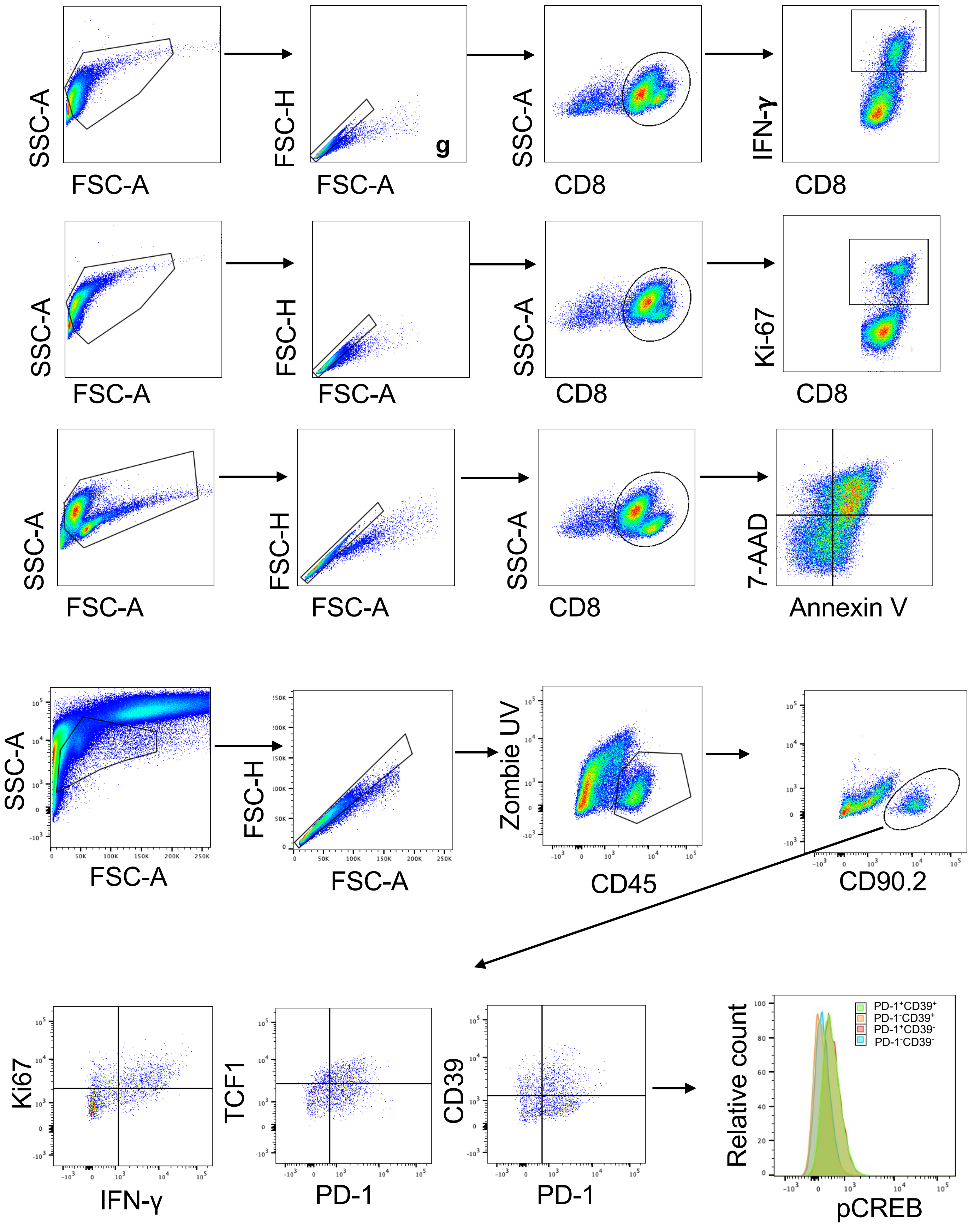

Figure S10. Gating strategy for chronic stimulation and in vivo experiments. (**a**) For chronic stimulation experiments, lymphocytes were gated from forward scatter area (FSC-A) and side scatter area (SSC-A). Single cells were distinguished from doublet cells in forward scatter height (FSC-H) and forward scatter area (FSC-A). Live CD8 cells were then gated. (**a**) For in vivo experiments, tumor infiltrating lymphocytes were gated from FSC-A and SSC-A. Single cells were distinguished from doublet cells in FSC-H and FSC-A. Live CD45 cells were then gated. Transferred T cells were distinguished by CD3 and CD90.2 positive, and followed by further immune characterization.
